# Dissection of quantitative trait loci governing root system architecture under aluminium toxicity stress conditions in *Brassica carinata*-derived *Brassica juncea* introgression lines

**DOI:** 10.1186/s12870-026-09439-0

**Published:** 2026-07-22

**Authors:** Shrusti Goud, Omkar Maharudra Limbalkar, Kanaka K. K., Annappa Naik H J, Vyankatesh Dhanraj Bagul, Prashant Vasisth, Naveen Singh, Kishor U. Tribhuvan, Santosh Kumar, Avinash Pandey, Vijai Pal Bhadana, Sujay Rakshit

**Affiliations:** 1 Indian Council of Agricultural Research (ICAR)- Indian Institute of Agricultural Biotechnology, Ranchi, Jharkhand India; 2 Division of Genetics, ICAR- Indian Agricultural Research Institute, New Delhi, India; 3 ICAR-Indian Agricultural Research Institute, Hazaribagh, Jharkhand India; 4 ICAR-Central Institute for Cotton Research, Nagpur, Maharashtra India

**Keywords:** Indian mustard, Genotyping by sequencing, Ethiopian mustard, Abiotic stress tolerance, Acid stress, Al toxicity

## Abstract

**Background:**

Aluminium (Al) toxicity in acidic soils is a major constraint limiting rapeseed–mustard productivity, particularly affecting *Brassica juncea* cultivation in acid-prone regions. The narrow genetic base of *B. juncea* restricts genetic improvement for Al toxicity stress tolerance. To enhance genetic variability and introgress beneficial genomic regions, *Brassica carinata*-derived *B. juncea* introgression lines (ILs) were developed and evaluated to dissect the genetic basis of root system architecture and stress tolerance under Al toxicity conditions.

**Results:**

Significant genetic variability was observed among the ILs for fourteen morpho-physiological traits evaluated under Al toxicity stress. Genotyping-by-sequencing generated 7,434 high-quality SNP markers, which were used to construct a high-density linkage map spanning 3,234.9 cM with an average marker interval of 2.3 cM. QTL analysis identified 24 significant QTLs, including 17 associated with root and shoot morphological traits and 7 linked to stress tolerance indices. Notable QTLs included *qRV.1B.1* for root volume and *qNF.3B.3* for number of forks, explaining 26.3% and 26.0% of phenotypic variation, respectively. Three QTL hotspots were detected, including a prominent region on chromosome 2B harboring QTLs for multiple stress tolerance indices. Several putative candidate genes with known roles in stress response pathways were identified within these QTL regions, including *COBRA-like* involved in cell wall expansion, peroxidase and *cytochrome P450* associated with oxidative detoxification, and *GATA*, *SAUR-like*, zinc-knuckle, and DNA damage-inducible proteins involved in stress signalling and regulation.

**Conclusions:**

This study elucidates the genetic architecture of Aluminium toxicity tolerance and identifies key QTLs and putative candidate genes governing root system architecture and stress adaptation in *Brassica juncea* introgression lines. The results demonstrate that interspecific introgression from *B. carinata* generated useful variation for root traits, enabling the identification of genomic regions associated with aluminium toxicity tolerance through GBS-based QTL mapping. These findings provide valuable genomic resources and promising targets for marker-assisted breeding to develop Aluminium toxicity-tolerant cultivars suitable for acidic soil environments.

**Supplementary Information:**

The online version contains supplementary material available at https://doi.org/10.1186/s12870-026-09439-0.

## Background

Rapeseed-mustard is an important group of edible oilseed crops cultivated worldwide, occupying approximately 42.48 million hectares (Mha) with a total production of about 86 million metric tons (MMT). It constitutes the third most important oilseed crop after soybean and oil palm [1]. In India, Indian mustard (*Brassica juncea* L. Czern & Coss) dominates within the rapeseed-mustard group of oilseeds, covering more than 90% of the total acreage (8.90 Mha) [2]. Despite its economic importance and high oil content, the productivity of Indian mustard is severely affected by several abiotic stresses, including aluminium (Al) toxicity under acid soils.

Improving the productivity and stress resilience of Indian mustard is essential for enhancing domestic edible oil production and reducing yield losses under adverse environmental conditions. With a view to achieve the self-sufficiency in edible oil, there is an urgent need to improve the productivity of Indian mustard. However, the narrow genetic base of this crop has severely constrained its genetic improvement, thereby limiting the scope for realizing higher genetic gains under adverse environmental conditions [3]. A substantial proportion of Indian mustard varieties have been developed from the cultivar Varuna or its derivatives [3–5], resulting in limited genetic diversity and impeding sustained crop improvement. This restricted genetic variability has limited the development of cultivars capable of maintaining stable productivity under abiotic stress conditions, including Al toxicity under acidic soil. Among the major abiotic stresses, soil acidity is a major constraint to mustard productivity in the north-eastern [6], eastern, and western regions of the country [7], rendering the crop particularly vulnerable to aluminium (Al) toxicity.

Acidic soils (pH < 5.5) cover nearly 30–40% of the world’s arable land and affect about 30.6 million hectares (Mha) in India, representing approximately 9.3% of the country’s total geographical area [7]. Under such conditions, soil pH below 5.5 results in the release of phytotoxic monomeric Al³⁺ ions, which readily enter root cells and inhibit cell division and elongation at the root apex. This leads to severe restriction of root growth and, consequently, reduced uptake of water and nutrients. Aluminium toxicity is therefore considered the major factor limiting crop productivity in acidic soils, with adverse effects observed even at micromolar concentrations within minutes of exposure [8, 9]. Although practices such as lime application and the use of phosphorus-based fertilizers can partially alleviate Al³⁺ toxicity, these methods are less effective in acidic subsoils and in aluminium-sensitive crops. Therefore, the development of aluminium toxicity tolerant cultivars remains the most practical and sustainable strategy for improving crop productivity under acidic soils conditions [10, 11].

Root architecture plays a vital role in plant adaptation to abiotic stresses. Since roots are the primary site of aluminium toxicity, root architectural traits provide valuable indicators of aluminium toxicity stress tolerance. Root-related traits play an important role in water and nutrient acquisition under stress conditions [12]. Hence, identification of genomic regions associated with root-related traits under Al toxicity stress could facilitate the development of tolerant cultivars through molecular breeding approaches.

The relatives of *Brassica* species are known to possess several desirable traits, including tolerance to drought, heat, cold and soil acidity [13–15]. Wide hybridization offers a novel approach to introduce these desirable traits into cultivated *Brassica* by creating novel genetic variability that can be further selected and utilized in breeding programs [16]. Ethiopian mustard (*Brassica carinata*; 2n = 4x = 34; BBCC) is known for its tolerance to several abiotic stresses, including drought, salinity and other adverse conditions [17–19]. Its sexual compatibility with Indian mustard (*B. juncea*) allows successful in vivo hybridization, making it a valuable donor for broadening the narrow genetic base of Indian mustard [20]. Introgression lines (ILs) developed through backcrossing and biparental mating represent powerful pre-breeding tools with reduced genetic background noise, enabling high-resolution mapping of quantitative trait loci (QTLs) with major and minor effects. Unlike conventional mapping populations, ILs contain defined donor genomic segments in a largely uniform genetic background, thereby improving the precision of QTL detection for complex traits. Since *B. carinata* possesses substantial variability for abiotic stress tolerance, these ILs provide a valuable resource for identifying genomic regions associated with Al toxicity tolerance in Indian mustard. Previous studies have shown that introgression of *B. carinata* genomic segments into *B. juncea* significantly improved seed yield and its related traits under abiotic stresses, including aluminium toxicity stress [5, 19, 21].

Aluminium toxicity tolerance is a polygenic, complex trait reported in several crop species, including mustard [22, 23]. To improve Al toxicity stress tolerance, breeding strategies such as introgression from wild relatives, quantitative trait loci (QTL) mapping, and marker-assisted backcross breeding (MABB) are widely used [24]. Among these, QTL mapping requires a large number of molecular markers distributed across the genome to achieve high-resolution mapping. Although simple sequence repeats (SSRs) have been commonly used for this purpose, their limited abundance and uneven genomic distribution restrict the high-resolution mapping of genomic regions. The development of next-generation sequencing (NGS) technologies has largely overcome these limitations by enabling the generation of high-density, genome-wide markers in the form of single-nucleotide polymorphisms (SNPs) or insertion-deletion (indels), thereby improving the resolution and efficiency of QTL detection.

NGS-based platforms provide a cost-effective means for genotyping large numbers of SNP sites, generating thousands to millions of markers across the genome. While whole-genome resequencing is highly effective when a reference genome is available, it remains expensive for crop species with large and complex genomes. As an alternative, genotyping-by-sequencing (GBS) has emerged as a cost-effective and high-throughput approach that allows simultaneous genome-wide SNP discovery and genotyping, making it well suited for high-resolution QTL mapping. This approach has been successfully applied for high-resolution linkage mapping, particularly in introgression lines developed from related species of *Brassica* [19, 25–27]. However, few studies have reported QTLs associated with Al toxicity stress tolerance in *B. napus* [23, 28]. In particular, information regarding QTLs governing aluminium toxicity tolerance in *B. juncea* is extremely limited. Furthermore, the genetic basis of Al toxicity tolerance in *B. carinata*-derived introgression lines of Indian mustard remains largely unexplored.

To enhance the ability of *Brassica juncea* to withstand abiotic stresses, efforts at the ICAR-Indian Agricultural Research Institute (ICAR-IARI), New Delhi, were made to broaden its genetic base through the development of introgression lines (ILs) carrying genomic segments from *Brassica carinata*. In the present study, these ILs were evaluated at ICAR-Indian Institute of Agricultural Biotechnology (ICAR-IIAB), Ranchi, under Al toxicity stress conditions. The present study aimed to identify QTLs and candidate genes associated with Al toxicity tolerance under acidic stress conditions. To the best of our knowledge, this is the first report in which *B. carinata*–derived introgression lines have been utilized for understanding the genetic basis of aluminium toxicity stress tolerance in Indian mustard. The findings of this study provide valuable insights into genomic regions associated with Al toxicity tolerance and may facilitate future marker-assisted breeding for the development of aluminium toxicity tolerant mustard cultivars.

## Materials and methods

### Development of introgression lines

Interspecific hybridization was employed to introgress genomic segments from *Brassica carinata* accession BC 4 into elite *Brassica juncea* cultivar DRMRIJ 31. This cultivar was developed under the ICAR-All India Coordinated Research Project on Rapeseed and Mustard and maintained in our breeding programme. DRMRIJ 31, commonly called Giriraj, was developed at ICAR-Indian Institute of Rapeseed and Mustard Research, Bharatpur, Rajasthan. A total of 80 *Brassica juncea* introgression lines (ILs) at the F_8_ generation, carrying genomic segments from *B. carinata*, were developed at ICAR-IARI, New Delhi [5]. These ILs originated from a cross between *B. juncea* cv. DRMRIJ 31 and *B. carinata* accession BC 4, followed by biparental mating among phenotypically selected plants in the F₂ population [19]. Subsequent phenotypic selection of desirable segregants exhibiting close resemblance to the recurrent *B. juncea* parent across successive filial generations resulted in the development of *B. carinata*–derived *B. juncea* ILs. The resulting ILs were confirmed to be homozygous and cytologically stable, as reported in our earlier study evaluating their heterotic expression [5]. Furthermore, molecular characterization of these ILs validated the presence of introgressed genomic segments from *B. carinata* [29]. The morphological, cytological, and fertility stability of ILs have already been validated [5].

### Phenotypic evaluation under Al toxicity stress

**Fig. 1:**
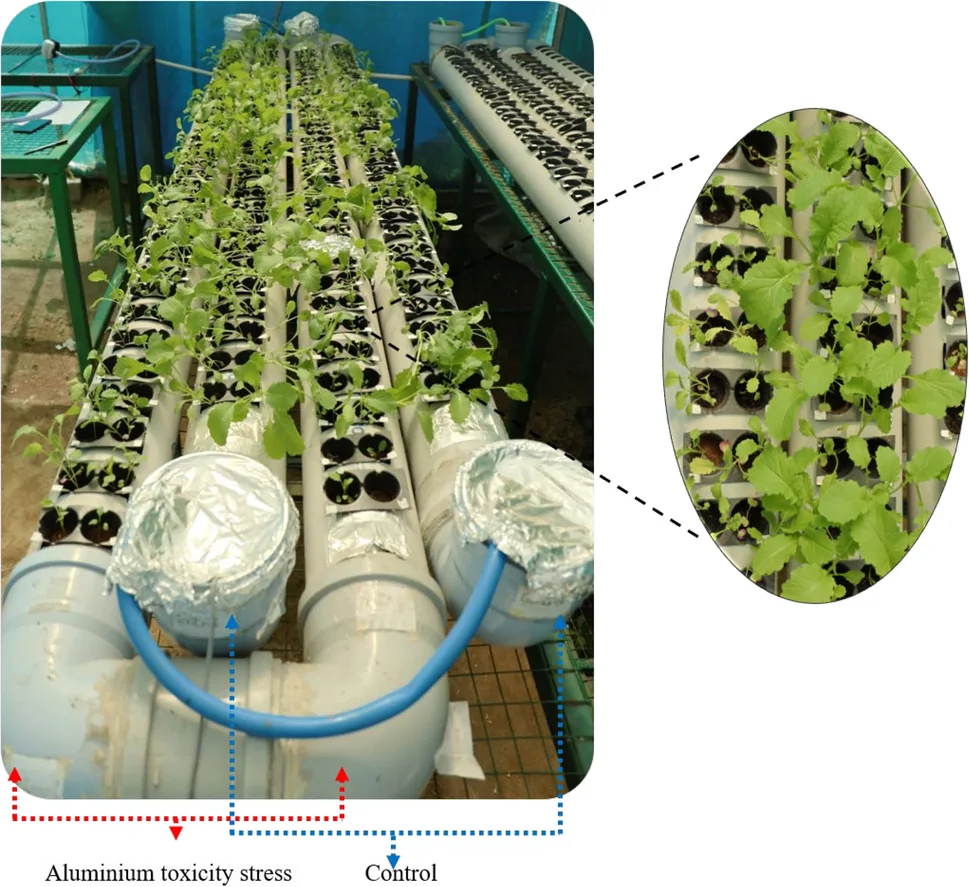
Custom-built hydroponic system at ICAR-IIAB, Ranchi, used for screening mustard genotypes under control and aluminium toxicity stress conditions

A set of eighty ILs along with two parental lines, viz., *B. juncea* cv. DRMRIJ 31 and *B. carinata* acc. BC 4 were evaluated under both control and aluminium toxicity stress conditions during *rabi* 2024–25 at ICAR-IIAB, Ranchi. Seeds were germinated in pro-trays filled with pre-wetted cocopeat. After 5–7 days of uniform growth, healthy seedlings were carefully transplanted into the pro-trays placed in the planting ports of the hydroponic setup. The hydroponic system was custom-built using PVC pipes with uniformly spaced rectangular openings designed to hold the pro-trays securely. Two independent U-shaped PVC pipelines were used to maintain separate nutrient circulation under control and Al toxicity stress conditions. A submersible pump facilitated continuous nutrient mixing and aeration in both control and Al toxicity stress conditions (Fig. 1). Each genotype was evaluated with four biological replicates within each treatment condition. Uniform nutrient circulation, continuous aeration, and standardized growth conditions were maintained throughout the experiment to minimize system-level variation.

Aluminium toxicity stress was imposed using a half-strength modified Hoagland’s nutrient solution supplemented with 200 µM AlCl₃·6 H₂O and adjusted to pH 4.3. In contrast, a half-strength Hoagland’s nutrient solution without Al³⁺, maintained at pH 6.8, served as the control. The acidic pH of the stress treatment was maintained to simulate acidic soil conditions associated with enhanced Al³⁺ solubility and bioavailability. However, since the control and stress treatments differed in both pH and aluminium concentration, the observed phenotypic responses may reflect the combined effects of acidic pH and aluminium toxicity. An acidic control without aluminium supplementation was not included in the present study. Half-strength Hoagland’s solution was selected as it closely approximates the ionic composition of natural soil solutions, thereby providing physiologically relevant growth conditions [30, 31]. The nutrient solution contained the following final concentrations (µmol L⁻¹): macronutrients—KH₂PO₄ (500), KNO₃ (2,500), Ca(NO₃)₂·4 H₂O (2,500), and MgSO₄·7 H₂O (1,000); and micronutrients—H₃BO₃ (23), MnCl₂·4 H₂O (4.7), ZnSO₄·7 H₂O (0.39), CuSO₄·5 H₂O (0.16), Na₂MoO₄·2 H₂O (0.41), and Fe-EDTA (45), prepared following the method originally described by Hoagland and Arnon (1940) [32]. To maintain nutrient stability, ionic balance and prevent microbial contamination, the nutrient solution was renewed every 10 days. Solution pH was monitored daily and adjusted using 0.1 M HCl or NaOH to ensure consistent aluminium bioavailability throughout the experimental period.

Each genotype was evaluated with four replications per treatment, and seedlings were grown for 21 days after transplanting under shade-net house conditions. Data were recorded for different morpho-physiological traits, including shoot length (SL), longest root length (LRL), chlorophyll index (CI) measured using a SPAD meter, shoot fresh weight (SFW), root fresh weight (RFW), and shoot and root dry weights (SDW and RDW). Data on SDW and RDW were recorded after oven-drying of samples at 65 ± 2 °C for 72 h until constant weight was achieved. Root morphological traits—total root length (TRL), root surface area (RSA), average root diameter (ARD), root volume (RV), number of root tips (NT), number of forks (NF), and number of crossings (NC)—were recorded using a root image analysis system. For root image acquisition, roots were gently washed with distilled water and arranged in a single, non-overlapping layer in a transparent tray containing a thin layer of water to minimize shadowing. Root images were captured using the WinRHIZOPro (version 2024a) system coupled with an Epson Expression 13000XL flatbed scanner at a resolution of 600 dpi. Image analysis was performed using WinRHIZOPro with a default grayscale threshold value of 128 for effective root–background separation. All samples were scanned and analyzed under identical parameters to ensure consistency and reproducibility.

### Statistical analyses

Phenotypic data for all morpho-physiological traits were subjected to analysis of variance following a Completely Randomized Design (CRD) statistical model,

$$Y{ij}=\mu+Gi=\varepsilon{ij}$$

Where Y*ij* is the observed value for the *i*th genotype in the *j*th replication; µ is the overall population mean; G*i* is the genotypic effect; and ε*ij* is the random error.

Phenotypic data under control and aluminium stress conditions were analysed separately using the above CRD model. Analysis of variance (ANOVA) was performed independently for each treatment condition to assess genotypic variation among the introgression lines. The relative response of genotypes to aluminium stress was further evaluated using stress tolerance indices derived from comparative trait performance under control and stress conditions.

Prior to ANOVA, residual normality and homogeneity of variance were assessed using Shapiro–Wilk and Levene’s tests, respectively. Trait distributions and potential outliers were further examined through boxplots and residual diagnostics. Since most traits satisfied ANOVA assumptions, no data transformation was required before statistical analysis.

Broad-sense heritability (H²) for morpho-physiological traits under control and aluminium stress conditions was estimated using variance components derived from ANOVA to assess the reliability of phenotypic variation among introgression lines.

Analysis was performed using IBM SPSS Statistics v. 26.0 [33] and R software v. 4.4.2 [34], with significance of genotypic differences tested at α = 0.05 and α = 0.01 levels using F-tests. Mean squares for genotypes and treatments were used to assess phenotypic variation among introgression lines under control and Al toxicity stress conditions. To visualize trait distributions and assess treatment differences, notched boxplots were generated in R software using ggplot2 package [35], with genotype-wise mean trait values, displaying medians, interquartile ranges (25^th^ − 75^th^ percentiles), and outliers marked as open circles with whiskers extending 1.5 times the interquartile range. Wilcoxon signed-rank tests were conducted to identify significant differences between control and Al toxicity stress conditions (*p* ≤ 0.05; *p* ≤ 0.01). In addition, means were overlaid as black diamond symbols, with vertical error bars representing ± standard error (SE), to provide a complementary estimate of central tendency and dispersion.

### Assessment of stress tolerance

The mean values of total root length (TRL) under control (Lc) and Al toxicity stress (Lt) were used to compute stress tolerance indices, including Root Tolerance Index (RTI), Tolerance (TOL), Percent Root Reduction (RR%), Stress Susceptibility Index (SSI), Mean Productivity (MP), Geometric Mean Productivity (GMP), Harmonic Mean (HM) and Stress Tolerance Index (STI) as per the formulae presented in Table 1. Total root length was selected for stress tolerance index calculations because it is widely recognized as one of the most sensitive indicators of aluminium stress response in plants. Moreover, in the present study, TRL exhibited positive and significant associations with other root architectural and biomass traits across introgression lines (Supplementary Fig. 1), suggesting that it effectively represented overall performance of plant under aluminium stress conditions. Genotypes were ranked for each index as well as based on their average rank across all indices, enabling objective comparison and classification of tolerance levels.

**Table 1:** Stress tolerance indices for total root length estimated in ILs evaluated under Al toxicity stress

SN	Index	Formula	Description	Reference
1.	Root Tolerance Index (RTI)	RTI =$\:\frac{{Net\:L}_{t}}{{Net\:L}_{c}}\times100$	Higher RTI indicates tolerant genotype	[36]
2.	Tolerance (TOL)	TOL =$\:{{L}_{c}-L}_{t}$	Lower TOL indicates tolerant genotype	[37]
3.	Percent Root Reduction (RR%)	RR% =$\:\frac{{{L}_{c}-L}_{t}}{{L}_{c}}\:\times100$	Lower RR% indicates tolerant genotype	[38]
4.	Stress Susceptibility Index (SSI)	SSI =$\:\frac{\mathrm{1-}\frac{{L}_{t}}{{L}_{c}}\text{}}{\mathrm{1-}\frac{\stackrel{-}{{\:L}_{t}}}{\stackrel{-}{{\:L}_{c}}}}$	Lower SSI indicates tolerant genotype	[37]
5.	Mean productivity (MP)	MP=$\:\frac{{Lc+L}_{t}}{2}$	Higher MP indicates better root development across both treatment conditions	[37]
6.	Geometric Mean Productivity (GMP)	GMP=$\:\sqrt{{Lc\times\:L}_{t}}$	High GMP indicates consistently good root growth under both control and stress	[37]
7.	Harmonic mean (HM)	HM =$\:\frac{{2(Lc\times\:L}_{t})}{{Lc+L}_{t}}$	Higher HM reflects stable and balanced root growth across stress and control	[37]
8.	Stress Tolerance Index (STI)	STI =$\:\:\frac{{(Lc\times\:L}_{t})}{\stackrel{-}{{L}_{c}}2}$	Higher STI denotes genotypes with strong root development under stress while maintaining good root length in control	[37]

Where,$\:{\:L}_{t}\:$*–* Total root length in Treatment,$\:{\:L}_{c}$*–* Total root length in Control,

$\:\stackrel{-}{{\:L}_{c}}$*–* Mean of Total root length (TRL) for the entire population under control conditions

$L_{t}$– Mean of total root length for the entire population under stress conditions

### Genotyping of introgression lines

A total of 80 introgression lines (ILs) were selected for genotyping-by-sequencing (GBS) analysis. Genomic DNA was extracted from each genotype using a modified cetyl trimethyl ammonium bromide (CTAB) method as described by Saghai-Maroof et al. (1984) [39]. Genotyping services were outsourced to NxGenBio Life Sciences Private Limited [19]. Sequencing was performed on the Illumina HiSeq 4000 platform using TruSeq kits, following the RAD-seq protocol of Elshire et al. (2011) [40] and Andrews et al. (2016) [41]. For demultiplexing, the FASTX Toolkit [42] was used, and read quality was checked with FastQC [43]; Fastp was used for adapter trimming and to remove reads with an average Phred score < 30 and read depth < 10. High-quality reads were aligned to the *Brassica juncea* cv. Varuna UDSC Var 1.1 reference genome [44] with BWA *v0.7.17* [45], and processed via SAMtools *v0.1.19* [46]. SNP and indel calling were performed using GATK *v3.6* [47] and BCFtools *v1.9* [48] in a consensus Haplotypecaller approach with ploidy adjustment for tetraploid species. Variants with quality score ≥ 30 were retained, SNPs were filtered to exclude monomorphic, missing and heterozygous loci in parents and to keep only those with MAF > 0.05 and missing rate < 10%. Linkage map construction used SNPs from the GBS data, retaining markers with MAF > 0.05 and a missing genotype rate < 0.01. Redundant SNPs were removed via QTL IciMapping Version 4.2 [49], binned by segregation with a distortion value threshold of 0.01, with the least-missing SNP from each bin selected. Markers were grouped, ordered and rippled using the Kosambi mapping function [50] in IciMapping 4.2 [49].

### Mapping of QTLs and candidate gene analysis

After filtering, the final set of non-redundant, polymorphic SNPs was grouped, ordered, and rippled using the Kosambi mapping function [50] in IciMapping 4.2 to generate a framework linkage map spanning the *B. juncea* A and B genomes. Quantitative trait loci (QTLs) conferring aluminium toxicity stress tolerance were mapped using phenotypic data from 80 introgression lines evaluated under both control and stress conditions, integrated with the GBS-based SNP genotyping data. QTL mapping was conducted using the BIP (bi-parental) function based on the ICIM (Inclusive Composite Interval Mapping) method implemented in IciMapping 4.2. Permutation testing (1,000 permutations at *p* = 0.05) was performed in IciMapping 4.2 to estimate empirical LOD thresholds for QTL detection. The observed permutation-based thresholds were close to 3.0 across traits. Based on the permutation results and commonly accepted standards used in QTL mapping studies, a LOD threshold of ≥ 3.0 was adopted for identification of putative QTLs. QTLs with LOD ≥ 3.0 and phenotypic variance explained (PVE) > 10% were retained as significant QTLs for further analysis. Identified QTLs were named following the nomenclature proposed by [51] and [52] with each QTL designated by the prefix “q” followed by a trait abbreviation and the corresponding linkage group identifier. Multiple QTLs on the same linkage group were numbered sequentially based on physical position. Candidate genes underlying QTL confidence intervals were identified by extracting FASTA nucleotide sequences corresponding to marker intervals from the *Brassica juncea* reference genome. The physical size of each QTL interval and the total number of predicted genes within each interval were recorded. The extracted sequences were subjected to gene prediction using the AUGUSTUS gene prediction tool [53], and predicted protein sequences were functionally annotated using InterProScan [54] integrated with the PANTHER database for domain and family classification. Because several QTL intervals contained a large number of predicted genes, candidate genes were prioritized based on functional annotation, reported involvement in abiotic stress adaptation pathways, and evidence from published literature related to aluminium toxicity response, oxidative stress regulation, ion transport, transcriptional regulation, root development, and cell wall modification. Representative biologically plausible candidate genes identified within significant QTL intervals were subsequently discussed.

## Results

### Phenotypic evaluation of introgression lines under aluminium stress

**Fig. 2:**
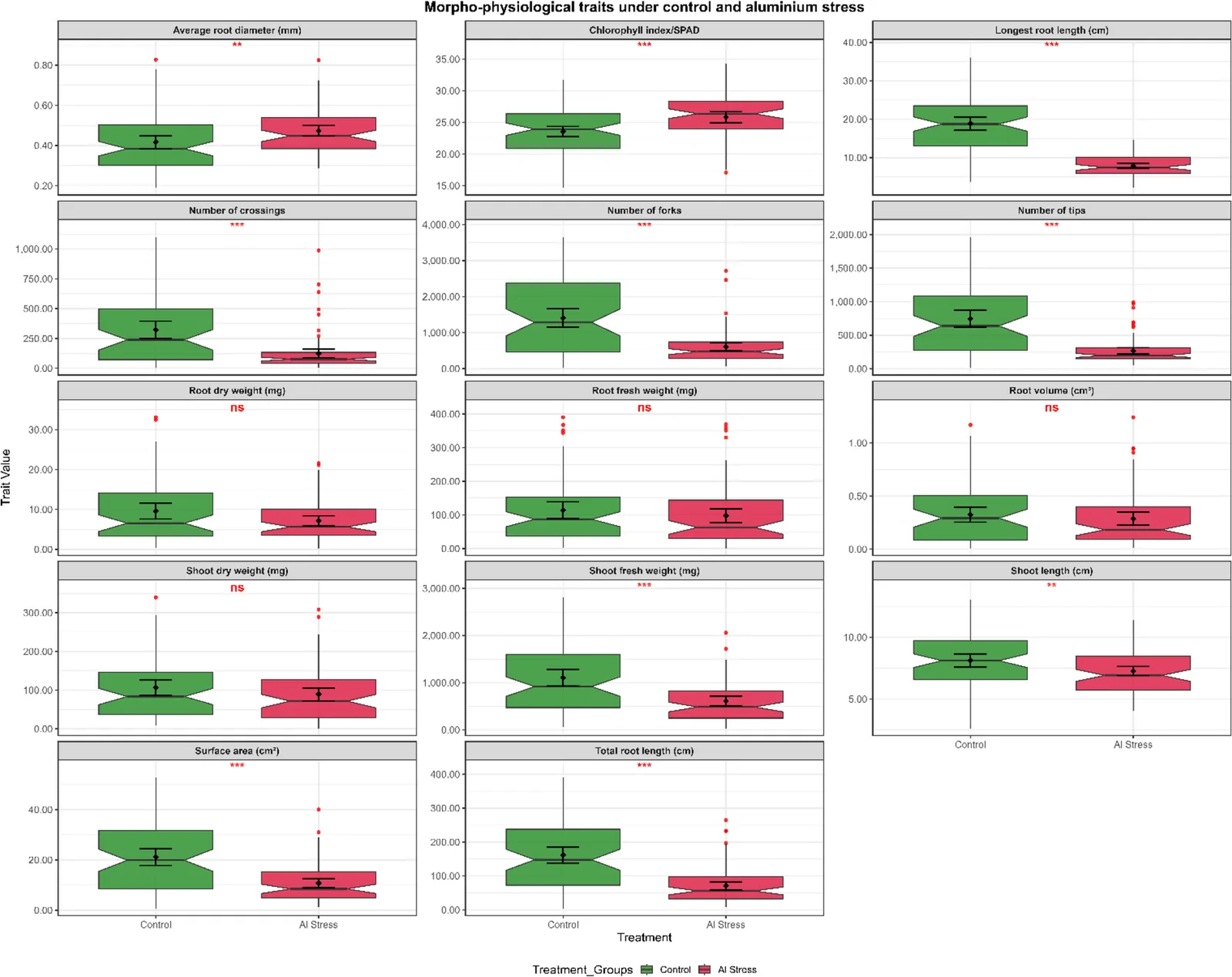
Notched Boxplots showing differences for morpho-physiological traits in ILs evaluated for control and Al stress under hydroponic conditions. Box edges represent upper and lower quartile, with median value shown as a bold line in the middle of the box. Whiskers represent 1.5 times the quartile of the data. Individuals falling outside the range of the whiskers shown as red open circles, and means were overlaid as black diamond symbols, standard error is shown with vertical error bars

Considerable phenotypic variation was observed among the introgression lines for all fourteen morpho-physiological traits evaluated under both control and aluminium toxicity stress conditions. The frequency distributions of all traits followed an approximately normal pattern (Fig. 2), suggesting quantitative inheritance of the studied traits. Notched boxplots comparing control and Al toxicity stress conditions for each morpho-physiological trait were analyzed using the Wilcoxon test, with significance levels indicated by *p*-values (Fig. 2). The analysis also revealed significant differences in mean performance between control and Al toxicity stress conditions for studied traits, viz., total root length, root surface area, average root diameter, root volume, number of root tips, number of forks, number of crossings, longest root length, shoot length, chlorophyll index, root fresh weight, shoot fresh weight, root dry weight and shoot dry weight. Notably, the mean values of average root diameter and chlorophyll index were significantly higher under Al toxicity stress conditions compared to control conditions (Fig. 2).

The analysis of variance revealed a highly significant phenotypic variation for all traits under both control and Al toxicity stress conditions (Table 2), indicating the presence of substantial genetic variability among the introgression lines. Several introgression lines exhibited transgressive segregation under Al toxicity stress conditions, surpassing both parental lines for key root architectural and biomass traits (Table 2).

**Table 2:** Analysis of variance of introgression lines (ILs) for different morpho-physiological traits evaluated under Control (C) and Treatment (T) conditions

			Source of Variation					Range	
			Mean Sum of Squares		Mean ± SE				
SN	Traits	Conditions	Treatments (df = 79)	Error(df = 240)	DRMRIJ 31	BC 4	ILs		H^2^
1	TRL	C	98967.48**	33842.57	409.69 ± 109.55	188.92 ± 63.05	208.31 ± 17.59	3.78–667.82	0.66
		T	12119.21**	6185.78	50.92 ± 17.36	56.38 ± 10.84	70.82 ± 6.15	9.10–264.67	0.63
2	SA	C	2685.62**	1220.52	59.67 ± 13.77	39.15 ± 17.19	29.00 ± 2.90	0.66–127.88	0.66
		T	109.05**	39.52	6.93 ± 1.26	9.65 ± 1.86	10.74 ± 0.90	1.22–40.07	0.72
3	ARD	C	0.10**	0.05	0.59 ± 0.08	0.67 ± 0.10	0.42 ± 0.02	0.19–0.85	0.64
		T	0.10**	0.06	0.53 ± 0.14	0.58 ± 0.07	0.50 ± 0.02	0.29–1.06	0.59
4	RV	C	7.62**	4.85	1.83 ± 1.02	0.35 ± 0.10	0.90 ± 0.15	0.01–8.79	0.57
		T	0.48**	0.21	0.11 ± 0.01	0.21 ± 0.08	0.32 ± 0.04	0.01–1.77	0.67
5	NT	C	3980191.60**	1,542,628	2020.00 ± 328.86	1289.88 ± 540.52	1089.19 ± 111.53	9.60–4830.50	0.70
		T	157449.78**	94467.35	198.88 ± 45.08	282.00 ± 65.13	267.33 ± 22.18	47.50–989.50	0.59
6	NF	C	17848996.00**	8,724,786	4541.12 ± 776.85	3227.75 ± 1346.22	2199.88 ± 236.17	15.53–9223.37	0.64
		T	916410.97**	430338.4	443.69 ± 83.18	753.19 ± 253.53	606.68 ± 53.51	59.17–2716.50	0.65
7	NC	C	4158873.00**	2,193,300	1433.00 ± 454.64	1135.12 ± 762.10	793.02 ± 114.00	1.67–4744.25	0.62
		T	102928.12**	49318.24	77.00 ± 20.76	128.94 ± 45.06	124.84 ± 17.93	4.67–987.50	0.65
8	LRL	C	268.75**	104.22	37.20 ± 7.74	21.27 ± 1.12	19.45 ± 0.92	3.66–41.70	0.70
		T	28.74*	21.21	9.76 ± 2.56	5.52 ± 0.62	7.85 ± 0.30	2.10–14.55	0.52
9	SL	C	21.96**	7.00	11.84 ± 0.95	11.76 ± 0.87	8.13 ± 0.26	2.63–13.03	0.74
		T	13.01**	5.43	7.93 ± 0.65	6.90 ± 0.62	7.36 ± 0.20	4.05–14.38	0.68
10	CL	C	271.32**	52.79	26.85 ± 1.82	31.04 ± 3.02	24.38 ± 0.92	14.65–89.70	0.83
		T	58.88**	21.64	27.90 ± 2.03	31.03 ± 2.59	25.83 ± 0.43	17.07–34.26	0.71
11	RFW	C	152839.77**	46,080	350.66 ± 27.96	168.42 ± 12.69	186.46 ± 21.85	1.71–881.38	0.75
		T	44467.37**	16,194	69.51 ± 2.68	66.21 ± 3.16	104.28 ± 11.79	0.96–594.26	0.71
12	SFW	C	5429725.80**	3,380,895	1850.12 ± 69.63	1385.94 ± 31.55	1382.54 ± 130.26	65.72–5425.91	0.58
		T	834465.99**	686,471	749.75 ± 38.62	409.04 ± 12.27	610.99 ± 51.07	30.68–2060.66	0.49
13	RDW	C	4490.81**	1366	18.99 ± 0.65	9.32 ± 0.20	20.12 ± 3.75	0.34–203.44	0.75
		T	2018.63**	1308	10.95 ± 0.39	13.42 ± 0.39	12.64 ± 2.51	0.27–154.03	0.56
14	SDW	C	206873.08**	91,856	170.26 ± 4.90	83.51 ± 1.46	195.61 ± 25.43	8.99–967.37	0.67
		T	22566.14**	14,232	130.32 ± 6.46	82.76 ± 5.92	89.24 ± 8.40	1.08–308.28	0.57

*TRL* Total root length, *RSA* root surface area, *ARD* Average root diameter, *RV* Root volume, *NT* Number of root tips, *NF* Number of forks, *NC* Number of crossings, *LRL *Longest root length, *SL *Shoot length, *CL* Chlorophyll index, *RFW* Root fresh weight, *SFW* Shoot fresh weight, *RDW* Root dry weight, *SDW* Shoot dry weight, *H*^2^ broad sense heritability, ** Significance *p* < 0.01; * *p* < 0.05

The mean performance of ILs was markedly superior to that of DRMRIJ 31 and BC 4. Specifically, ILs recorded a significantly greater total root length (70.82 cm) compared to DRMRIJ 31 (50.92 cm) and BC 4 (56.38 cm). Similarly, surface area was higher in ILs (10.74 cm²) relative to DRMRIJ 31 (6.93 cm²) and BC 4 (9.65 cm²). Enhanced root volume was also observed in ILs (0.32 cm³), exceeding both DRMRIJ 31 (0.11 cm³) and BC 4 (0.21 cm³). In addition, root fresh weight was substantially higher in ILs (104.28 g) compared with DRMRIJ 31 (69.51 g) and BC 4 (66.21 g). Under control conditions, transgressive segregation among ILs was observed for root dry weight and shoot dry weight (Table 2).

Under control conditions, total root length (TRL) varied widely, ranging from 3.78 to 667.82 cm with a mean of 208.31 ± 17.59 cm. Exposure of ILs to Al toxicity stress markedly reduced TRL, which ranged from 9.10 to 264.67 cm with a mean of 70.82 ± 6.15 cm, representing an approximate 66% reduction compared to control conditions. Aluminium toxicity also caused substantial declines in other root architectural traits, including root surface area (63% reduction, from 29.00 to 10.74 cm²), number of forks (72% reduction, from 2199.88 to 606.68), number of root tips (75% reduction, from 1089.19 to 267.33), and number of crossings (84% reduction, from 793.02 to 124.84). Collectively, these pronounced reductions highlight the strong inhibitory effect of Al toxicity on root system development, proliferation, and branching complexity.

Moderate to high broad-sense heritability estimates were observed for almost all studied traits under both control and aluminium stress conditions, indicating reliable phenotypic differentiation among introgression lines and suitability of the dataset for QTL mapping.

### Stress tolerance indices

Stress tolerance indices, including Root Tolerance Index (RTI), tolerance (TOL), percent root reduction (RR%), stress susceptibility index (SSI), mean productivity (MP), geometric mean productivity (GMP), harmonic mean (HM), and stress tolerance index (STI), were estimated for total root length in the introgression lines evaluated under aluminium toxicity stress and control conditions (Supplementary Table 1). The root tolerance index (RTI) ranged from 6.64 (IL139) to 499.75 (IL138), while tolerance (TOL) varied widely from − 82.12 (IL138) to 400.36 (IL161), indicating substantial genetic variability in stress response. Percent root reduction (RR%) ranged from − 399.75 to 93.36%, and stress susceptibility index (SSI) ranged from − 5.40 to 1.26, reflecting differential sensitivity among ILs. Negative values observed for RR% and SSI in certain ILs indicate that some genotypes maintained or exhibited relatively improved root growth under stress conditions compared to control conditions, reflecting differential stress adaptation responses among introgression lines. Based on stress tolerance indices, several ILs exhibited superior performance, particularly IL154, IL161, IL157, IL155, IL97, IL146, and IL144, which recorded high values of mean productivity (MP), geometric mean productivity (GMP), harmonic mean (HM), and stress tolerance index (STI). Among these, IL154 showed the highest STI (1.08), followed by IL161 (0.98) and IL157 (0.84), indicating their superior tolerance and stable performance under stress and control conditions. In contrast, sensitive genotypes such as IL81, IL118, and IL123 exhibited low MP, GMP, HM, and STI values.

Pearson’s correlation analysis revealed significant associations among several root architectural traits and stress tolerance indices under aluminium toxicity stress conditions (Supplementary Fig. 1). Strong positive correlations were observed among major root growth traits including total root length, root surface area, number of root tips, number of forks, root fresh weight, and shoot fresh weight. Among the stress tolerance indices, very strong relationships among RTI, RR%, SSI, and RRG (Supplementary Fig. 1), reflecting their derivation from common root growth measurements under control and stress conditions.

### Construction of high-density linkage map

Genotyping-by-sequencing (GBS) generated a large volume of high-quality sequence data for the parental genotypes and representative introgression lines (Table 3). The total number of reads per genotype ranged from 13.35 million (IL100) to 121.89 million (IL136), indicating adequate sequencing depth across samples. A high proportion of reads was successfully aligned to the reference genome, with mapped reads varying from 13.17 million to 120.27 million. The mapping efficiency was consistently high across all genotypes, ranging from 94.60% (BC 4) to 98.78% (DRMRIJ 31), reflecting the good quality of sequencing data and reliable genome alignment (Table 3). The alignment statistics of representative introgression lines along with parental genotypes were presented in Table 3, while detailed alignment statistics for all introgression lines were provided in the Supplementary Table 3. The uniformly high mapping rates among parents and ILs ensured robust SNP discovery and provided a strong foundation for the construction of a high-density linkage map.

**Table 3:** Genotyping by sequencing alignment statistics for parental genotypes and representative introgression lines (ILs) for sequencing quality assessment

SN	Genotype*	Total Reads	Mapped Reads	Mapping Rate
1	BC4	30,50,012	28,85,184	94.60%
2	DRMRIJ-31	36,04,624	35,60,713	98.78%
3	IL84	51,09,658	50,16,224	98.17%
4	IL87	54,35,076	53,66,436	98.74%
5	IL89	37,86,324	37,48,645	99.00%
6	IL100	13,35,170	13,17,259	98.66%
7	IL103	13,40,672	13,28,225	99.07%
8	IL108	13,31,514	13,18,801	99.05%
9	IL115	37,54,018	37,15,668	98.98%
10	IL123	62,89,862	62,22,674	98.93%
11	IL136	1,21,88,724	1,20,26,993	98.67%
12	IL138	13,31,126	13,02,500	97.85%

*All 80 ILs were included in the GBS analysis and subsequent QTL mapping

A high-density linkage map was constructed using 7,434 high-quality SNP markers distributed across 18 chromosomes of *Brassica juncea*, derived from introgression lines generated by crossing DRMRIJ 31 and *B. carinata* accession BC 4 (Table 4). These markers covered a total map length of 3,234.87 cM with an average marker interval of 2.30 cM. The A genome harbored 2,513 markers (33.80%) across 10 chromosomes spanning 1,686.06 cM, with an average marker density of 1.49 per cM. In contrast, the B genome showed dense distribution, accommodating 4,921 markers (66.20%) across 8 chromosomes with a total length of 1,548.82 cM and a marker density of 3.18 per cM. The highest number of SNPs were observed on chromosome 1B (813 SNPs, 10.94%), followed by 3B (707 SNPs, 9.51%) and 4B (666 SNPs, 8.96%) (Table 4). Marker density varied across chromosomes, ranging from 0.62 per cM (10 A) to 4.81 markers per cM (1B). Among the two genomes of *Brassica juncea*, the B genome exhibited higher marker density, accounting for 66.20% of the total markers with an average spacing of 3.18 cM per marker, compared to the A genome, which contained 33.80% of the markers with an average marker interval of 1.49 cM (Table 4).

**Table 4:** Chromosome-wise SNP marker statistics

SN	Chromosome	Number of SNPs	% Markers	Map length (cM)	Marker density
1.	1 A	455	6.12	157.62	2.89
2.	2 A	254	3.42	138.12	1.84
3.	3 A	295	3.97	248.12	1.19
4.	4 A	224	3.01	83.48	2.68
5.	5 A	237	3.19	189.19	1.25
6.	6 A	166	2.23	154.96	1.07
7.	7 A	208	2.80	198.42	1.05
8.	8 A	134	1.80	144.67	0.93
9.	9 A	425	5.72	187.01	2.27
10.	10 A	115	1.55	184.46	0.62
11.	1B	813	10.94	169.04	4.81
12.	2B	514	6.91	148.03	3.47
13.	3B	707	9.51	268.46	2.63
14.	4B	666	8.96	212.44	3.13
15.	5B	535	7.20	198.53	2.69
16.	6B	452	6.08	161.01	2.81
17.	7B	582	7.83	250.91	2.32
18.	8B	652	8.77	140.39	4.64
19.	A genome	2513	33.80	1686.06	1.49
20.	B genome	4921	66.20	1548.82	3.18
	Total	7434		3234.87	2.30

### Mapping of QTLs for morpho-physiological traits under Al toxicity stress

QTL mapping under aluminium toxicity stress conditions revealed several significant loci associated with different traits under study. A total of 24 significant QTLs were identified across multiple chromosomes, with phenotypic variance explained (PVE) ranging from 10.08% to 26.26%. Out of 24 significant QTLs identified, seventeen were associated with morpho-physiological traits across chromosomes 1B, 2A, 2B, 3A, 3B, 4B, 6A, 6B, 7A, 7B, 8B and 9A, with PVE ranging from 10.10% to 26.26%. A QTL for root surface area, *qSA.6B.1*, was mapped on chromosome 6B, explaining 16.09% of phenotypic variation (Table 5). Two QTLs governing average root diameter, *qARD.6 A.1* and *qARD.1B.1*, were mapped on chromosomes 6 A and 1B with PVE values of 17.26% and 13.13%, respectively. Two QTLs for root volume were identified, *qRV.1B.1* on chromosome 1B with the highest PVE of 26.26% and *qRV.4B.1* on 4B with PVE of 16.00%. A significant locus *qNF.3B.3* governing the number of forks was mapped on chromosome 3B with PVE of 26.02%, while three QTLs for the number of crossings were mapped across chromosomes 3A, 3B and 8B. Among them, *qNC.3B.1* had the highest LOD of 20.04 and PVE 22.43% (Table 5).

**Table 5:** List of significant QTLs identified for different morpho-physiological traits evaluated under Al toxicity stress conditions

SN	Trait	QTL	Chr.	Pos.	Flanking Markers	LOD	PVE (%)	Add	Marker Interval(cM)
1.	Surface area	*qSA.6B.1*	6B	128	*6B_15059700–6B_15144213*	7.67	16.09	6.65	127.5–128.5
2.	Average root diameter	*qARD.6A.1*	6A	80	*6A_36966305–6A_34816179*	14.85	17.26	0.10	77.5–80.5
3.	Average root diameter	*qARD.1B.1*	1B	120	*1B_19790453–1B_19979059*	12.26	13.13	0.09	119.5–120.5
4.	Root volume	*qRV.1B.1*	1B	128	*1B_17653981–1B_16999532*	5.66	26.26	0.24	126.5–129.5
5.	Root volume	*qRV.4B.1*	4B	114	*4B_1697703–4B_4660764*	4.90	16.00	0.21	112.5–114.5
6.	Number of tips	*qNT.9A.1*	9A	149	*9A_45743661–9A_45737915*	4.27	10.73	108.12	148.5–149.5
7.	Number of tips	*qNT.7B.1*	7B	62	*7B_43476486–7B_48930379*	4.88	13.73	150.94	60.5–64.5
8.	Number of forks	*qNF.3B.3*	3B	250	*3B_6503214–3B_6672912*	12.56	26.02	1272.21	249.5–250.5
9.	Number of crossings	*qNC.3A.1*	3A	115	*3A_22586128–3A_23619571*	14.12	12.60	240.59	114.5–115.5
10.	Number of crossings	*qNC.3B.1*	3B	250	*3B_6503214–3B_6672912*	20.04	22.43	451.04	249.5–250.5
11.	Number of crossings	*qNC.8B.1*	8B	93	*8B_36611003–8B_52931757*	15.83	14.67	213.31	92.5–93.5
12.	Longest root length	*qLRL.2 A.1*	2A	136	*2A_14336283–2A_4589590*	5.14	14.31	-2.63	135.5–137.5
13.	Longest root length	*qLRL.7A.1*	7A	96	*7A_14692208–7A_19334660*	4.11	11.97	-1.44	95.5–96.5
14.	Longest root length	*qLRL.2B.1*	2B	22	*2B_4484775–2B_58676910*	5.84	16.55	-1.54	21.5–27.5
15.	Root fresh weight	*qRFW.2A.1*	2A	96	*2A_17510339–2A_32002031*	3.94	10.10	62.64	90.5–100.5
16.	Shoot dry weight	*qSDW.6B.1*	6B	55	*6B_13861974–6B_14560639*	3.0102	15.8764	-61.385	47.5–65.5
17.	Root dry weight	*qRDW.3B.1*	3B	250	*3B_6503214–3B_6672912*	51.10	17.85	-73.32	249.5–250.5

where, *Chr* Chromosome, *Pos* Chromosome position (cM) of the QTL, *PVE* Phenotypic variance explained (%), *Add* Additive effect (positive values of the additive effect indicated that alleles from parent ‘BC 4’ were in the direction of increasing trait score and negative indicated that alleles from parent ‘DRMRIJ 31’ were in the direction of increasing trait score)

### Mapping of QTLs for stress tolerance indices

Seven significant QTLs associated with aluminium toxicity tolerance indices were identified on chromosomes 2B, 6A and 9A, explaining phenotypic variation ranging from 10.08% to 22.12%. A significant QTL hotspot was observed on chromosome 2B at 146 cM, harboring four co-localized QTLs for critical stress tolerance indices viz., root tolerance index (*qRTI.2B.2*), percent root reduction (*qRR.2B.2*), stress susceptibility index (*qSSI.2B.2*) and relative root growth (*qRRG.2B.2*) under Al toxicity stress (Table 6). These loci exhibited highly significant LOD scores of 17.41 and each explained approximately 13.29 to 13.30% of phenotypic variance. Two QTLs associated with geometric mean productivity (*qGMP.6A.1*) and harmonic mean (*qHM.6A.1*) were mapped on chromosome 6A with 22.12% and 21.84% of phenotypic variation, respectively (Table 6).

**Table 6:** List of significant QTLs detected for different stress tolerance indices under control and Al toxicity stress conditions

SN	Trait	QTL	Chr.	Pos	Flanking Markers	LOD	PVE (%)	Add	Marker Interval (cM)
1.	Root tolerance index	*qRTI.2B.2*	2B	146	*2B_1271491–2B_10786746*	17.41	13.29	138.05	145.5–146.5
2.	Tolerance	*qTOL.9 A.1*	9 A	144	*9A_12155171–9A_13961289*	3.15	10.08	101.75	143.5–144.5
3.	Percent root reduction	*qRR.2B.2*	2B	146	*2B_1271491–2B_10786746*	17.41	13.30	-138.05	145.5–146.5
4.	Stress susceptibility index	*qSSI.2B.2*	2B	146	*2B_1271491–2B_10786746*	17.41	13.30	-1.87	145.5–146.5
5.	Relative root growth	*qRRG.2B.2*	2B	146	*2B_1271491–2B_10786746*	17.41	13.29	5.30	145.5–146.5
6.	Geometric mean productivity	*qGMP.6 A.1*	6 A	29	*6A_37988602–6A_35399730*	5.96	22.12	40.31	27.5–31.5
7.	Harmonic mean	*qHM.6 A.1*	6 A	29	*6A_37988602–6A_35399730*	4.13	21.84	29.83	26.5–30.5

Where, *Chr* Chromosome, *Pos* Chromosome position (cM) of the QTL, *PVE* Phenotypic variance explained (%), *Add* Additive effect (positive values of the additive effect indicated that alleles from parent ‘BC 4’ were in the direction of increasing trait score and negative indicated that alleles from parent ‘DRMRIJ 31’ were in the direction of increasing trait score

### Identification of co-localized QTLs and candidate gene analysis

**Fig. 3:**
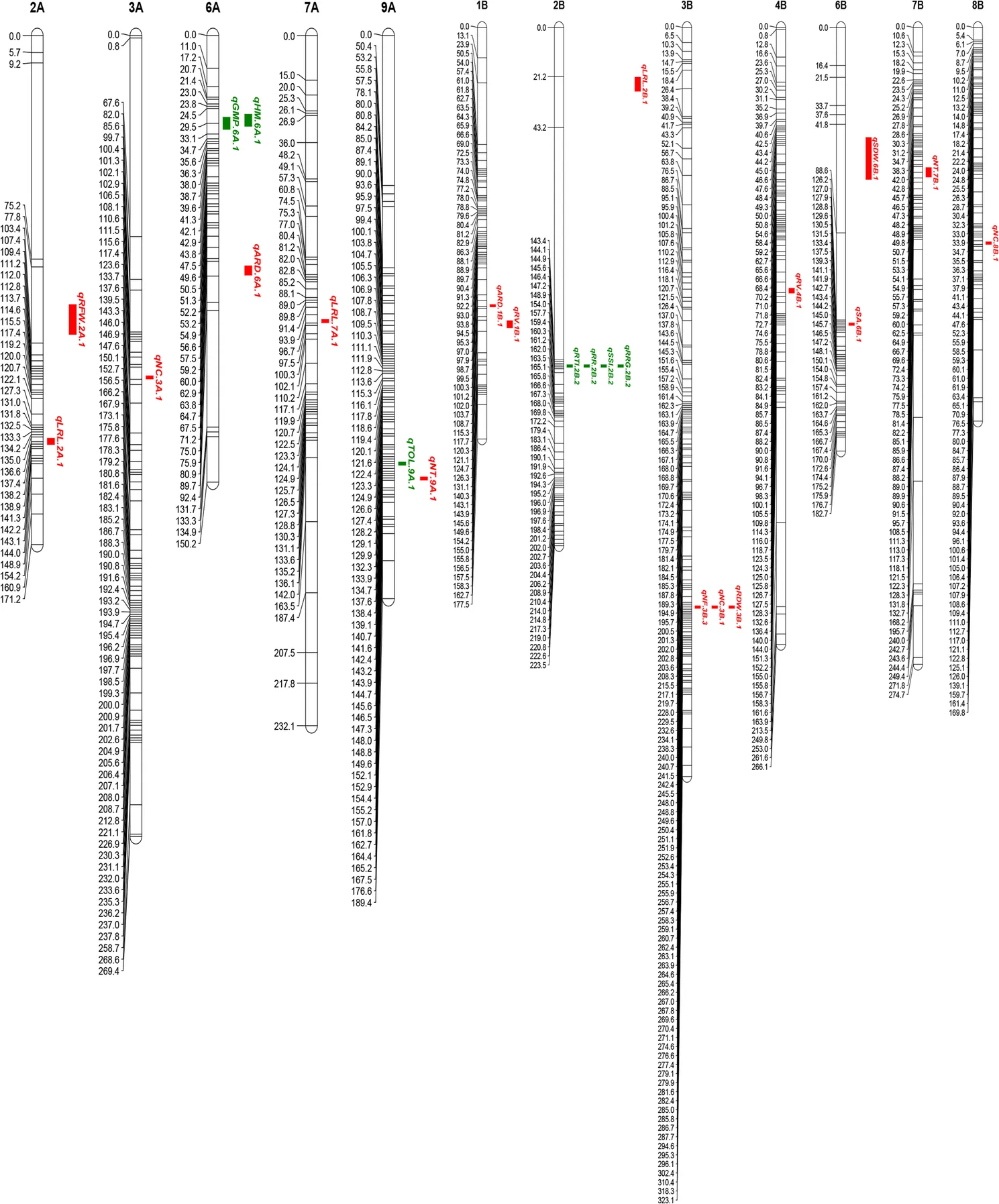
The linkage map of ILs derived from *B. juncea* cv. DRMRIJ 31 x *B. carinata* acc. BC 4 depicting QTLs for different morpho-physiological traits and aluminium toxicity stress tolerance indices

Analysis of QTL co-localization identified several genomic regions controlling two or more morpho-physiological traits, stress tolerance indices, and root-related traits under Al toxicity stress (Table 7; Fig. 3). Three significant QTL hotspots were identified where multiple QTLs for different traits were co-localized within the same chromosomal intervals. A significant co-localized QTL region on chromosome 6 A at 29 cM (confidence interval: 26.5–31.5 cM) harbored QTLs for geometric mean productivity (*qGMP.6A.1*) and harmonic mean productivity (*qHM.6A.1*), explaining 22.12% and 21.84% of the phenotypic variance, respectively, with high LOD scores (Table 7). This region contained candidate genes encoding *zinc knuckle (CCHC-type)* proteins and *DNA damage-inducible protein-like* proteins, which are known to be associated with stress responses. Another prominent co-localized QTL cluster was detected on chromosome 2B at 146 cM (confidence interval: 145.5–146.5 cM), governing root tolerance index (*qRTI.2B.2*), percent root reduction (*qRR.2B.2*), stress susceptibility index (*qSSI.2B.2*), and relative root growth (*qRRG.2B.2*). These QTLs showed high LOD values (≥ 17.4) and explained approximately 13.29–13.30% of the phenotypic variation, indicating a promising genomic region influencing aluminium stress tolerance. Candidate genes within this region included *peroxidase-related* proteins, *GATA* transcription factors, and *cytochrome P450*, which are implicated in oxidative stress mitigation and root growth regulation.

**Table 7:** List of co-localized significant QTLs for one or more traits and position of the candidate genes in the identified QTL regions

SN	Trait	QTL	Chr.	Marker Interval	Position (cM)	Confidence Interval (cM)	LOD	PVE (%)	Candidate genes reported
1	Geometric mean productivity	*qGMP.6A.1*	6A	*6A_37988602–6A_35399730*	29	27.5–31.5	5.96	22.12	*Zinc knuckle (CCHC-type) family* proteins [55, 56], *DNA Damage-Inducible Protein 1-like* [57].
2	Harmonic mean	*qHM.6A.1*			29	26.5–30.5	4.13	21.84	
3	Root tolerance index	*qRTI.2B.2*	2B	*2B_1271491–2B_10786746*	146	145.5–146.5	17.41	13.29	*Peroxidase 72-Related* [58]; *GATA Transcription Factor* [59]; *Cytochrome P450* [59].
4	Percent root reduction	*qRR.2B.2*			146	145.5–146.5	17.41	13.30	
5	Stress susceptibility index	*qSSI.2B.2*			146	145.5–146.5	17.41	13.30	
6	Relative root growth	*qRRG.2B.2*			146	145.5–146.5	17.41	13.29	
7	Number of forks	*qNF.3B.3*	3B	*3B_6503214–3B_6672912*	250	249.5–250.5	12.56	26.02	*Glycosyl hydrolases* [60]; *E3 ubiquitin-protein ligase RNF13-like* [61, 62].

A third significant QTL cluster was identified on chromosome 3B at 250 cM (confidence interval: 249.5–250.5 cM), associated with number of forks (*qNF.3B.3*), number of crossings (*qNC.3B.1*), and root dry weight (*qRDW.3B.1*). These QTLs exhibited high phenotypic variance explained (17.85–26.02%) and strong LOD scores, suggesting their importance in regulating root system architecture under Al stress. Candidate genes in this region encoded *glycosyl hydrolases*,* ubiquitin-protein ligases*, and *NF13*-like proteins, which are known to play roles in cell wall remodeling, protein turnover, and stress adaptation.

In silico candidate gene analysis within significant QTL regions identified functionally relevant genes involved in abiotic stress tolerance (Table 7; Supplementary Table 2). The QTL hotspot on chromosome 2B at 146 cM harbored genes encoding *peroxidase 72* and *cytochrome P450* genes involved in oxidative stress detoxification and *GATA* transcription factors regulating growth responses. The chromosome 6A region at 29 cM contained *zinc knuckle (CCHC-type*) family proteins and *DNA damage-inducible protein 1-like* genes contributing to transcriptional regulation and DNA repair under abiotic stresses. Similarly, on chromosome 3B, genes encoding glycosyl hydrolases and *E3 ubiquitin-protein ligase RNF13-like* were identified, linked with cell wall remodeling and protein turnover.

## Discussion

Aluminium (Al) toxicity in acidic soils is a major constraint to crop productivity worldwide and poses a significant limitation to *Brassica juncea* cultivation in acid-prone regions where soil pH falls below 5.5 and exchangeable Al³⁺ reaches phytotoxic levels [63–65]. The primary effect of Al toxicity is the root apex, where inhibition of cell elongation and division leads to a reduction in root elongation, impaired nutrient uptake, and ultimately reduced biomass and yield [63, 64]. Improving tolerance to Al toxicity stress, therefore, requires a detailed understanding of the genetic mechanisms governing tolerance to Al toxicity stress. The tolerance to Al toxicity stress in crop plants, including *Brassicas*, is governed by multiple quantitative traits controlled by polygenes [23, 28]. Polygenes interact with environmental factors and exhibit continuous phenotypic variation, complicating direct phenotypic selection in conventional breeding [66, 67]. Marker-assisted selection (MAS) provides an effective alternative by enabling the selection of favourable alleles underlying stress tolerance irrespective of environmental variation, but its success depends on the prior identification of molecular markers tightly linked to quantitative trait loci (QTLs) controlling aluminium toxicity tolerance [65]. High-density genetic linkage maps constructed with abundant single nucleotide polymorphism (SNP) markers are essential for reliable QTL detection and effective MAS, as demonstrated in other crops where dense SNP scans have identified Al toxicity tolerance QTLs [68]. In the present study, a genotyping-by-sequencing (GBS) approach was employed to generate genome-wide SNPs and construct a high-density linkage map spanning both A and B genomes of *B. juncea*, providing a robust genomic foundation for subsequent QTL mapping of aluminium toxicity stress tolerance.

Interspecific hybridization has been effectively used to broaden the genetic base of cultivated *Brassica* species through introgression of novel alleles from related species [69]. Given its resilience to multiple abiotic stresses, *Brassica carinata* serves as a valuable donor for improving *B. juncea* [20, 29]. In this study, previously developed *B. carinata*-derived *B. juncea* introgression lines were evaluated under Al toxicity stress [5, 19, 21]. A genotyping-by-sequencing (GBS) approach was used to generate genome-wide SNP markers and construct a high-density linkage map across the A and B genomes to identify QTLs/genes associated with aluminium tolerance.

### Phenotypic variation and aluminium stress response in introgression lines

Analysis of variance revealed highly significant genetic variation (*p* < 0.001) among the ILs for all morpho-physiological traits under both control and aluminium (Al) stress conditions (Table 2). This wide phenotypic dispersion confirms that the IL population captures substantial genetic variability, an essential prerequisite for reliable QTL detection and high-resolution genomic mapping.

Under control conditions, total root length (TRL) in ILs ranged from 3.78 to 667.82 cm (mean = 208.31 ± 17.59 cm), whereas under Al toxicity stress, it reduced drastically to 9.10–264.67 cm with a mean of 70.82 ± 6.15 cm, representing a 66% reduction (Table 2). Root growth inhibition is widely recognized as one of the earliest and most sensitive indicators of Al toxicity, primarily due to rapid Al³⁺ accumulation in the root apoplast and meristematic zones, which disrupts cell division, cell wall extensibility, and nutrient transport [8]. Similar suppression of root elongation has been reported in *Brassica napus* and other *Brassica* species under comparable Al exposure, although the magnitude of reduction varies depending on genotype, Al concentration, and duration of stress [70, 71].

Beyond primary root length, aluminium stress markedly reduced overall root system architecture. Surface area declined by 63% (29.00 to 10.74 cm²), number of forks by 72% (2199.88 to 606.68), number of root tips by 75% (1089.19 to 267.33), and crossings by 84% (793.02 to 124.84), collectively reflecting severe impairment of lateral root initiation and branching (Table 2). Such root architectural modifications likely result from Al-induced damage to root meristematic tissues and disruption of hormonal signalling pathways, particularly auxin and ethylene, which regulate lateral root development [8, 65]. The reduction in branching traits suggests that aluminium toxicity not only restricts root elongation but also compromises the absorptive capacity and exploratory efficiency of the root system. In the present study, aluminium stress was imposed under acidic pH conditions to simulate acid soil-associated Al toxicity commonly encountered under field environments. However, because the control and stress treatments differed in both pH and aluminium concentration, the observed phenotypic responses may partly reflect the combined effects of acidic pH and aluminium toxicity. Future studies incorporating acidic controls without aluminium supplementation would help to further distinguish pH-mediated and aluminium-specific effects on root development and stress tolerance.

Overall, the substantial phenotypic variation observed among ILs under Al toxicity stress highlights the quantitative and polygenic nature of tolerance mechanisms in *B. juncea* (Table 2; Fig. 2). The strong stress-induced differentiation among lines provides a robust phenotypic foundation for identifying genomic regions associated with tolerance to Al toxicity stress.

### Stress tolerance indices

The wide variation observed among the introgression lines for stress tolerance indices, viz., RTI, TOL, RR%, SSI, MP, GMP, HM, and STI, indicates the presence of substantial genetic variability for aluminium toxicity tolerance (Supplementary Table 1), which is expected in IL populations derived from interspecific introgression. Stress tolerance indices such as GMP, HM, and STI are considered more reliable selection criteria because they identify genotypes with stable performance under both stress and non-stress conditions [72]. In the present study, IL-154, IL-161, IL-157, IL-155, IL-97, IL-146, and IL-144 exhibited high STI, GMP, and HM values, indicating their ability to maintain better root growth and overall performance under aluminium stress. The superior performance of these ILs suggests successful introgression of favourable alleles governing aluminium tolerance from the donor parent into the genetic background of the recurrent parent. In contrast, genotypes with low STI and productivity indices and high SSI values were more susceptible, reflecting their inability to withstand stress-induced growth reduction. Similar findings have been reported where higher STI and related indices were associated with improved stress tolerance and yield stability [73, 74]. The occurrence of negative TOL and SSI values in some ILs further indicates their relatively better performance under stress, reflecting efficient stress adaptation mechanisms. The superior performance of several ILs compared to the parental lines confirms the effectiveness of introgression breeding in enhancing abiotic stress tolerance and has been widely reported in *Brassica* and other crops [8]. These tolerant ILs may serve as valuable genetic resources for breeding aluminium toxicity tolerant mustard cultivars and for identifying associated genomic regions.

### Construction of a high-density linkage map and QTL mapping for aluminium toxicity tolerance

Combining genotyping-by-sequencing (GBS) with biparental mapping has emerged as a powerful strategy for constructing high-density linkage maps and dissecting complex traits such as aluminium toxicity tolerance [75]. In the present study, the SNP-based linkage map comprising 7,434 markers spanning 3,234.87 cM with an average marker interval of 2.30 cM provided a robust genomic framework for high-resolution QTL detection. The higher marker density observed in the B genome (3.18 markers cM⁻¹) compared with the A genome (1.49 markers cM⁻¹) reflects effective introgression of genetic variation from *B. carinata*, highlighting the utility of interspecific hybridization for the transfer of novel alleles for stress adaptation into cultivated species. While SNP-based linkage maps have been widely employed for mapping agro-morphological traits in *Brassica* species [19], the present study represents one of the first applications of a GBS-derived high-density map developed from *B. carinata*-derived *B. juncea* introgression lines for QTL mapping under aluminium toxicity stress. This genomic resource provides a strong foundation for marker-assisted breeding aimed at developing aluminium-tolerant mustard cultivars.

A total of 24 QTLs were identified in the present study. Among these, 17 QTLs exhibited significant additive effects and explained variation in different morpho-physiological traits, whereas the remaining 7 QTLs were associated with stress tolerance indices (Tables 5 and 6). The results indicated that favorable alleles at 17 of the detected QTLs were derived from *B. carinata* acc. BC 4, accounting for 70.83% of the total QTLs detected. These QTLs regulated seven morpho-physiological traits and five stress tolerance indices, suggesting that alleles derived from *B. carinata* played a predominant role in enhancing aluminium (Al) toxicity tolerance in these ILs. In contrast, favorable alleles at the remaining seven QTLs were contributed by *B. juncea* (29.17%). This finding underscores that most favourable loci governing morpho-physiological traits under Al toxicity stress were contributed by the *B. carinata* acc. BC 4 in the ILs.

Out of the 17 QTLs identified in the IL population, 12 (approx. 71% of the total QTLs detected) were contributed by the B genome of *B. carinata*, while the remaining five (approx. 29%) were derived from the A genome. This pattern likely reflects preferential pairing and recombination between the homologous B genomes of the two species, along with recombination events between the A and C genomes, facilitating the introgression of genomic regions from *B. carinata* into *B. juncea* [19, 69, 76]. The present study revealed a higher number of QTLs associated with Al toxicity stress tolerance in the B genome, which is consistent with previous findings reporting a greater frequency of introgressed segments in the B genome compared to the A genome in the ILs [19, 29]. This enrichment may be attributed to the retention of novel alleles conferring Al toxicity tolerance in *B. carinata* [77], whereas some of these alleles may have been lost in *B. juncea* during evolution. Such genomic differentiation could also be a consequence of nucleo-cytoplasmic interactions that have led to substantial modifications in the B genome of *B. juncea*, while the B genome of *B. carinata* has largely remained conserved [78, 79].

In the current study, QTL analysis under Al toxicity stress revealed a strong genetic basis for root and morpho-physiological traits, leading to the identification of 24 significant QTLs distributed across multiple chromosomes, with phenotypic variance explained (PVE) ranging from 10.08% to 26.26% (Tables 5 and 6). Such a wide distribution of QTLs indicates a complex and polygenic genetic architecture governing Al stress tolerance, consistent with earlier reports demonstrating the involvement of multiple loci in crop responses to aluminium toxicity [28, 80, 81]. Notably, 17 significant QTLs associated with morpho-physiological traits were mapped on chromosomes 1B, 2 A, 2B, 3 A, 3B, 4B, 6 A, 6B, 7 A, 7B, 8B, and 9 A, further underscoring the polygenic nature of aluminium tolerance. Similar multi-locus control has been reported in rapeseed–mustard crops under Al stress conditions [23, 28]. The locus *qSA.6B.1*, explaining 16.09% of the variation in root surface area, and *qRV.1B.1*, accounting for 26.26% PVE for root volume, highlight the presence of strong genetic determinants influencing root architecture—an essential adaptive trait for nutrient uptake under Al toxicity stress. Additionally, root diameter QTLs *qARD.6 A.1* and *qARD.1B.1* emphasize the importance of structural root traits in conferring Al toxicity stress tolerance, as root morphological traits are widely recognized as reliable indicators of Al stress responses in various crop species, including mustard [23, 28]. The identification of *qNF.3B.3* associated with the number of root forks, along with *qNC.3B.1* governing the number of crossings, suggests that fine-scale root branching patterns are also under significant genetic control under toxic Al conditions. These findings align with meta-QTL analyses in crop plants that have identified clusters of Al-responsive loci, reinforcing the complexity and evolutionary conservation of Al toxicity tolerance mechanisms across cereals and oilseed crops [82]. Collectively, the results of the present study provide valuable genomic targets for marker-assisted selection aimed at enhancing aluminium tolerance through improvement of root system architecture. Similarly, seven significant QTLs associated with Al stress tolerance indices were identified. Notably, four of these QTLs were clustered on chromosome 2B (*qRTI.2B.2*,* qRR.2B.2*,* qSSI.2B.2*, and *qRRG.2B.2*), each exhibiting a high LOD score and explaining approximately 13.30% of the phenotypic variance. The co-localization of these loci within the same genomic region suggests either pleiotropic effects of a single significant locus or tight linkage among stress-responsive genes governing tolerance to Al toxicity stress conditions (Tables 5 and 6). In addition, *qGMP.6 A.1* (22.12% PVE) and *qHM.6 A.1* (21.84% PVE) mapped on chromosome 6 A, indicating that key loci responsible for maintaining stable performance across both control and Al stress conditions are concentrated in discrete genomic regions.

The present study was conducted under controlled hydroponic conditions during a single crop season and at a single location. Therefore, the identified QTLs should be considered as promising genomic regions associated with root system architecture responses under aluminium stress conditions. Further validation across multiple years, environments, larger populations, and field conditions will be necessary to confirm the stability, consistency, and breeding utility of these loci before their deployment in marker-assisted breeding programs.

### Identification of co-localized QTL and candidate genes in the QTL regions

Genomic regions harbouring multiple QTLs for distinct traits, commonly referred to as QTL hotspots, enable the simultaneous improvement of multiple traits and thereby accelerate breeding progress through marker-assisted selection (MAS) [83]. In the present study, three such co-localized QTL regions were identified. The first significant QTL hotspot was located on chromosome 2B, comprising *qRTI.2B.2*, *qRR.2B.2*, *qSSI.2B.2*, and *qRRG.2B.2* (Table 7; Fig. 3; Supplementary Table 2). This region harbors several putative candidate genes including, *COBRA-like* genes potentially involved in cell wall biosynthesis and aluminium ion exclusion, *peroxidase 72* and *cytochrome P450* enzymes associated with reactive oxygen species (ROS) scavenging and antioxidant biosynthesis, and *GATA* transcription factors implicated in abiotic stress-responsive gene regulation. Supporting evidence from Pesic et al. (2024) in *Miscanthus × giganteus* demonstrated upregulation of *COBRA-like* genes under heavy metal stress to reinforce cell wall integrity [84]. Similarly, *peroxidases* and *cytochrome P450* enzymes have been reported to play central roles in metal toxicity tolerance through ROS detoxification and phytohormone metabolism across multiple crops [58, 85]. Furthermore, *GATA* transcription factors in rice are rapidly induced under abiotic stress and coordinate downstream defense responses [59]. The correlation analysis further revealed strong interrelationships among several stress tolerance indices derived from root performance under control and stress conditions (Supplementary Fig. 1). As several of these indices are derived from root performance under control and stress conditions, their co-localization within this hotspot may partly reflect mathematically and biologically related phenotypes. Nevertheless, the possibility of pleiotropic effects or tight linkage among the underlying loci cannot be excluded.

A second hotspot was identified on chromosome 3B, where three QTLs governing root architectural traits co-localized: *qNF.3B.3* (26.02% PVE), *qNC.3B.1* (22.43% PVE), and *qRDW.3B.1* (17.85% PVE). This region contains putative candidate genes including, *glycosyl hydrolases* potentially involved in cell wall remodelling during lateral root emergence and *E3 ubiquitin-protein ligase RNF13-like* proteins that may mediate selective protein degradation for growth reprogramming under stress conditions. *Glycosyl hydrolase* upregulation has been shown to facilitate cell wall loosening during lateral root initiation under aluminium stress [60]. Furthermore, *E3 ubiquitin ligases* regulate root development under stress conditions through targeted degradation of growth-suppressor proteins [61, 62].

Additionally, a third hotspot was identified on chromosome 6 A, associated with stress tolerance indices including *qGMP.6 A.1* (22.12% PVE) and *qHM.6 A.1* (21.84% PVE), which reflect stable performance across control and stress environments. This genomic region harbours putative candidate genes including, *zinc knuckle CCHC-type* transcription factors and *DNA damage-inducible protein 1-like* (*DDI1-like*) genes, suggesting their possible involvement in stress-responsive regulatory networks. Zinc knuckle proteins are known to function as transcriptional regulators involved in cellular stress signaling, and CCHC-type zinc knuckle proteins have been shown to modulate transcriptional responses under stress-induced cellular damage in crop plants [55, 56]. Similarly, *DDI1-like* genes enhance DNA repair capacity under genotoxic stress conditions [57]. The co-localization of these plausible candidate genes within the identified QTL interval suggests their potential involvement in aluminium stress response mechanisms.

The clustering of significant-effect QTLs within three discrete genomic regions, each enriched with functionally complementary candidate genes, suggests that Al toxicity tolerance in *B. juncea* may involve coordinated molecular mechanisms related to oxidative stress detoxification (chromosome 2B), root architectural plasticity (chromosome 3B), and maintenance of genomic stability (chromosome 6 A). These QTL hotspots represent promising genomic regions for marker-assisted breeding and provide a useful foundation for future studies aimed at elucidating the genetic basis of aluminium toxicity tolerance in Indian mustard. However, validation of these loci in independent populations and under acidic soil field conditions will be necessary before their direct deployment in marker-assisted breeding programmes.

Although these candidate genes are biologically plausible based on functional annotation and previous literature reports, their roles in aluminium toxicity tolerance remain putative and require further validation through gene expression profiling and functional characterization studies.

## Conclusion

This study demonstrated the potential of *B. carinata*-derived *B. juncea* introgression lines for improving aluminium toxicity tolerance in *B. juncea* and provided critical insights into the genetic basis of root system architecture and stress tolerance traits under aluminium toxicity stress conditions. The *B. carinata*-derived introgression lines exhibited substantial variation for root architectural and biomass traits under aluminium stress, demonstrating the effectiveness of interspecific introgression in broadening the genetic base of *B. juncea*. This variation enabled the construction of a high-density GBS-based linkage map and the identification of genomic regions associated with aluminium toxicity tolerance. Twenty-four significant QTLs associated with morpho-physiological traits and stress tolerance indices were identified, with most favorable alleles originating from the B genome of *B. carinata*. QTL hotspots associated with multiple traits were identified in the present study, offering promising targets for marker-assisted selection and the pyramiding of favourable alleles. In silico analysis identified several putative candidate genes associated with stress-responsive pathways within the identified QTL regions, providing preliminary functional insights into the genetic basis of Al toxicity tolerance. The genetic resources and information generated in this study underscore the effectiveness of interspecific hybridization among *Brassica* species in broadening the genetic base and creating exploitable novel genetic variability. Collectively, these findings lay a strong foundation for the development of high-yielding cultivars with enhanced tolerance to aluminium toxicity in acid-prone regions. However, the identified QTLs and candidate genomic regions require further validation across multiple environments, years, independent populations, and acidic soil field conditions before their effective deployment in marker-assisted breeding programmes for aluminium toxicity tolerance in Indian mustard.

## Supplementary Information


Supplementary Material 1.


## Data Availability

The datasets generated and/or analysed during the current study are available in the NCBI Sequence Read Archive (SRA) repository under BioProject accession number PRJNA1023751, PRJNA1024079 and PRJNA1449767.
