# Supplementary material for: Dissection of quantitative trait loci governing root system architecture under aluminium toxicity stress conditions in Brassica carinata-derived Brassica juncea introgression lines
Source: BMC Plant Biol. 2026 Jul 22;26:1487. doi: 10.1186/s12870-026-09439-0 (PMC13523352; doi:10.1186/s12870-026-09439-0)
Supplement: Supplementary file 1 [file 12870_2026_9439_MOESM1_ESM.docx]

**Supplementary Table** **1**. Stress tolerance indices for total root length estimated in ILs along with their parents evaluated under Al toxicity stress and control conditions

| **IL** | **RTI** | **TOL** | **RR%** | **SSI** | **MP** | **GMP** | **HM** | **STI** |
| --- | --- | --- | --- | --- | --- | --- | --- | --- |
| IL79 | 23.03 | 142.78 | 76.97 | 1.04 | 114.11 | 89.02 | 69.45 | 0.16 |
| IL80 | 41.79 | 114.78 | 58.21 | 0.79 | 139.81 | 127.48 | 116.25 | 0.34 |
| IL81 | 9.29 | 149.39 | 90.71 | 1.23 | 89.99 | 50.20 | 28.00 | 0.05 |
| IL82 | 22.93 | 164.47 | 77.07 | 1.04 | 131.17 | 102.19 | 79.61 | 0.22 |
| IL83 | 8.03 | 285.66 | 91.97 | 1.24 | 167.78 | 88.04 | 46.19 | 0.16 |
| IL84 | 8.14 | 299.94 | 91.86 | 1.24 | 176.56 | 93.18 | 49.18 | 0.18 |
| IL85 | 19.12 | 248.85 | 80.88 | 1.09 | 183.27 | 134.56 | 98.80 | 0.37 |
| IL86 | 22.98 | 225.86 | 77.02 | 1.04 | 180.33 | 140.60 | 109.62 | 0.41 |
| IL87 | 393.63 | -18.88 | -293.63 | -3.97 | 15.87 | 12.76 | 10.26 | 0.00 |
| IL88 | 38.33 | 149.82 | 61.67 | 0.83 | 168.04 | 150.42 | 134.65 | 0.47 |
| IL89 | 114.97 | -8.48 | -14.97 | -0.20 | 60.90 | 60.76 | 60.61 | 0.08 |
| IL90 | 25.23 | 101.90 | 74.77 | 1.01 | 85.33 | 68.45 | 54.91 | 0.10 |
| IL91 | 25.34 | 106.52 | 74.66 | 1.01 | 89.42 | 71.83 | 57.70 | 0.11 |
| IL92 | 19.74 | 230.96 | 80.26 | 1.09 | 172.29 | 127.86 | 94.89 | 0.34 |
| IL93 | 50.63 | 40.52 | 49.37 | 0.67 | 61.82 | 58.40 | 55.18 | 0.07 |
| IL94 | 71.87 | 13.91 | 28.13 | 0.38 | 42.49 | 41.92 | 41.35 | 0.04 |
| IL95 | 23.50 | 29.64 | 76.50 | 1.03 | 23.93 | 18.78 | 14.74 | 0.01 |
| IL96 | 18.39 | 121.15 | 81.61 | 1.10 | 87.88 | 63.66 | 46.12 | 0.08 |
| IL97 | 23.60 | 307.54 | 76.40 | 1.03 | 248.75 | 195.53 | 153.70 | 0.79 |
| IL98 | 12.41 | 368.28 | 87.59 | 1.18 | 236.32 | 148.12 | 92.84 | 0.45 |
| IL99 | 9.62 | 344.28 | 90.38 | 1.22 | 208.78 | 118.14 | 66.85 | 0.29 |
| IL100 | 280.77 | -6.83 | -180.77 | -2.44 | 7.19 | 6.33 | 5.57 | 0.00 |
| IL101 | 57.52 | 50.72 | 42.48 | 0.57 | 94.03 | 90.55 | 87.19 | 0.17 |
| IL102 | 11.29 | 207.01 | 88.71 | 1.20 | 129.84 | 78.39 | 47.32 | 0.13 |
| IL103 | 26.04 | 176.02 | 73.96 | 1.00 | 149.98 | 121.45 | 98.34 | 0.30 |
| IL104 | 27.70 | 203.99 | 72.30 | 0.98 | 180.16 | 148.51 | 122.43 | 0.46 |
| IL105 | 18.33 | 191.65 | 81.67 | 1.10 | 138.84 | 100.47 | 72.71 | 0.21 |
| IL106 | 35.25 | 142.00 | 64.75 | 0.88 | 148.30 | 130.20 | 114.31 | 0.35 |
| IL107 | 23.31 | 180.16 | 76.69 | 1.04 | 144.84 | 113.42 | 88.81 | 0.27 |
| IL108 | 19.82 | 169.64 | 80.18 | 1.08 | 126.75 | 94.19 | 69.99 | 0.18 |
| IL109 | 13.41 | 210.37 | 86.59 | 1.17 | 137.76 | 88.96 | 57.45 | 0.16 |
| IL110 | 12.23 | 254.24 | 87.77 | 1.19 | 162.56 | 101.32 | 63.15 | 0.21 |
| IL111 | 116.57 | -9.68 | -16.57 | -0.22 | 63.25 | 63.07 | 62.88 | 0.08 |
| IL112 | 45.59 | 32.14 | 54.41 | 0.74 | 43.00 | 39.89 | 37.00 | 0.03 |
| IL113 | 11.60 | 209.67 | 88.40 | 1.20 | 132.34 | 80.77 | 49.29 | 0.13 |
| IL114 | 15.39 | 146.93 | 84.61 | 1.14 | 100.18 | 68.12 | 46.32 | 0.10 |
| IL115 | 21.90 | 46.24 | 78.10 | 1.06 | 36.09 | 27.71 | 21.28 | 0.02 |
| IL116 | 50.65 | 71.46 | 49.35 | 0.67 | 109.07 | 103.05 | 97.37 | 0.22 |
| IL117 | 11.17 | 134.30 | 88.83 | 1.20 | 84.04 | 50.54 | 30.39 | 0.05 |
| IL118 | 8.82 | 105.83 | 91.18 | 1.23 | 63.15 | 34.47 | 18.82 | 0.02 |
| IL119 | 126.23 | -11.58 | -26.23 | -0.35 | 49.95 | 49.61 | 49.28 | 0.05 |
| IL120 | 11.42 | 149.75 | 88.58 | 1.20 | 94.17 | 57.12 | 34.64 | 0.07 |
| IL121 | 57.20 | 27.72 | 42.80 | 0.58 | 50.91 | 48.99 | 47.14 | 0.05 |
| IL122 | 46.62 | 54.20 | 53.38 | 0.72 | 74.43 | 69.32 | 64.56 | 0.10 |
| IL123 | 11.33 | 103.20 | 88.67 | 1.20 | 64.78 | 39.17 | 23.68 | 0.03 |
| IL124 | 28.03 | 78.00 | 71.97 | 0.97 | 69.38 | 57.38 | 47.45 | 0.07 |
| IL126 | 100.24 | -0.15 | -0.24 | 0.00 | 61.88 | 61.88 | 61.88 | 0.08 |
| IL127 | 86.06 | 10.40 | 13.94 | 0.19 | 69.43 | 69.23 | 69.04 | 0.10 |
| IL128 | 122.64 | -8.01 | -22.64 | -0.31 | 39.37 | 39.16 | 38.96 | 0.03 |
| IL129 | 101.34 | -1.05 | -1.34 | -0.02 | 79.05 | 79.05 | 79.05 | 0.13 |
| IL130 | 24.33 | 96.14 | 75.67 | 1.02 | 78.98 | 62.67 | 49.73 | 0.08 |
| IL131 | 15.78 | 59.68 | 84.22 | 1.14 | 41.02 | 28.15 | 19.32 | 0.02 |
| IL132 | 66.47 | 50.11 | 33.53 | 0.45 | 124.40 | 121.85 | 119.35 | 0.31 |
| IL133 | 99.52 | 0.31 | 0.48 | 0.01 | 63.98 | 63.98 | 63.98 | 0.08 |
| IL134 | 30.85 | 102.00 | 69.15 | 0.93 | 96.50 | 81.93 | 69.55 | 0.14 |
| IL135 | 67.11 | 41.62 | 32.89 | 0.44 | 105.74 | 103.67 | 101.64 | 0.22 |
| IL136 | 153.56 | -28.36 | -53.56 | -0.72 | 67.12 | 65.61 | 64.13 | 0.09 |
| IL137 | 104.94 | -5.27 | -4.94 | -0.07 | 109.41 | 109.38 | 109.35 | 0.25 |
| IL138 | 499.75 | -82.12 | -399.75 | -5.40 | 61.60 | 45.92 | 34.23 | 0.04 |
| IL139 | 6.64 | 397.46 | 93.36 | 1.26 | 226.98 | 109.66 | 52.98 | 0.25 |
| IL140 | 26.62 | 101.81 | 73.38 | 0.99 | 87.83 | 71.58 | 58.33 | 0.11 |
| IL141 | 70.10 | 21.54 | 29.90 | 0.40 | 61.26 | 60.31 | 59.37 | 0.08 |
| IL142 | 107.35 | -3.52 | -7.35 | -0.10 | 49.66 | 49.63 | 49.59 | 0.05 |
| IL143 | 24.53 | 195.21 | 75.47 | 1.02 | 161.05 | 128.10 | 101.89 | 0.34 |
| IL144 | 34.69 | 187.87 | 65.31 | 0.88 | 193.71 | 169.41 | 148.16 | 0.59 |
| IL145 | 44.83 | 81.60 | 55.17 | 0.75 | 107.10 | 99.02 | 91.56 | 0.20 |
| IL146 | 28.36 | 230.53 | 71.64 | 0.97 | 206.53 | 171.37 | 142.20 | 0.61 |
| IL147 | 84.95 | 17.11 | 15.05 | 0.20 | 105.20 | 104.85 | 104.50 | 0.23 |
| IL149 | 46.03 | 95.30 | 53.97 | 0.73 | 128.92 | 119.79 | 111.31 | 0.30 |
| IL151 | 77.84 | 38.11 | 22.16 | 0.30 | 152.87 | 151.68 | 150.50 | 0.48 |
| IL152 | 35.57 | 133.71 | 64.43 | 0.87 | 140.66 | 123.76 | 108.89 | 0.32 |
| IL153 | 16.38 | 348.31 | 83.62 | 1.13 | 242.40 | 168.60 | 117.28 | 0.59 |
| IL154 | 25.25 | 339.86 | 74.75 | 1.01 | 284.73 | 228.46 | 183.31 | 1.08 |
| IL155 | 20.24 | 345.23 | 79.76 | 1.08 | 260.21 | 194.71 | 145.69 | 0.78 |
| IL156 | 39.50 | 124.54 | 60.50 | 0.82 | 143.57 | 129.36 | 116.56 | 0.35 |
| IL157 | 25.65 | 295.59 | 74.35 | 1.01 | 249.77 | 201.35 | 162.31 | 0.84 |
| IL159 | 52.56 | 89.12 | 47.44 | 0.64 | 143.30 | 136.20 | 129.44 | 0.38 |
| IL160 | 24.47 | 261.51 | 75.53 | 1.02 | 215.48 | 171.27 | 136.13 | 0.61 |
| IL161 | 19.26 | 400.36 | 80.74 | 1.09 | 295.69 | 217.62 | 160.16 | 0.98 |
| IL163 | 26.82 | 255.09 | 73.18 | 0.99 | 221.02 | 180.51 | 147.42 | 0.67 |
| DRMRIJ 31 | 13.58 | 324.16 | 86.42 | 1.17 | 213.00 | 138.20 | 89.66 | 0.39 |
| BC4 | 29.84 | 132.54 | 70.16 | 0.95 | 122.65 | 103.21 | 86.85 | 0.22 |

RTI= Root Tolerance Index; TOL= Tolerance (TOL); RR%= Percent Root Reduction; SSI= Stress Susceptibility Index; MP= Mean Productivity; GMP= Geometric Mean Productivity; HM= Harmonic Mean and STI= Stress Tolerance Index; Negative RR% and SSI values indicate relatively higher root growth under stress compared to control conditions.

**Supplementary Table 2.** Putative candidate genes identified from co-localized QTL intervals associated with stress tolerance indices and morpho-physiological traits under aluminium toxicity stress conditions.

| **SN** | **QTL name** | **Flanking markers** | **Physical  interval (Mb)** | **Confidence interval (cM)** | **Names of predicted genes in the QTL interval (No)** | **Prioritized candidate genes** |
| --- | --- | --- | --- | --- | --- | --- |
| 1 | *qGMP.6A.1;  qHM.6A.1* | *6A_37988602–6A_35399730* | 35.40–37.99  (2.59 Mb) | 27.5–31.5; 26.5–30.5 | ZINC KNUCKLE (CCHC-TYPE) FAMILY PROTEIN | Zinc knuckle (CCHC-type) family proteins [86-87];  DNA Damage-Inducible Protein 1-like [88]. |
|  |  |  |  |  | ZINC KNUCKLE (CCHC-TYPE) FAMILY PROTEIN |  |
|  |  |  |  |  | TRANSPOSASE, PUTATIVE-RELATED |  |
|  |  |  |  |  | GAG-POL-RELATED RETROTRANSPOSON |  |
|  |  |  |  |  | GAG-POL-RELATED RETROTRANSPOSON |  |
|  |  |  |  |  | GAG-POL-RELATED RETROTRANSPOSON |  |
|  |  |  |  |  | RIBONUCLEASE H |  |
|  |  |  |  |  | GAG-POL-RELATED RETROTRANSPOSON |  |
|  |  |  |  |  | TRANSPOSASE, PUTATIVE-RELATED |  |
|  |  |  |  |  | TRANSPOSASE, PUTATIVE-RELATED |  |
|  |  |  |  |  | GAG-POL-RELATED RETROTRANSPOSON |  |
|  |  |  |  |  | TRANSPOSASE, PUTATIVE-RELATED |  |
|  |  |  |  |  | TRANSPOSASE, PUTATIVE-RELATED |  |
|  |  |  |  |  | ZINC KNUCKLE (CCHC-TYPE) FAMILY PROTEIN |  |
|  |  |  |  |  | GAG-POL-RELATED RETROTRANSPOSON |  |
|  |  |  |  |  | POLYPROTEIN, PUTATIVE-RELATED |  |
|  |  |  |  |  | GAG-POL-RELATED RETROTRANSPOSON |  |
|  |  |  |  |  | GAG-POL-RELATED RETROTRANSPOSON |  |
|  |  |  |  |  | GAG-POL-RELATED RETROTRANSPOSON |  |
|  |  |  |  |  | TRANSPOSASE, PUTATIVE-RELATED |  |
|  |  |  |  |  | GAG-POL-RELATED RETROTRANSPOSON |  |
|  |  |  |  |  | TRANSPOSASE, PUTATIVE-RELATED |  |
|  |  |  |  |  | GAG-POL-RELATED RETROTRANSPOSON |  |
|  |  |  |  |  | GAG-POL-RELATED RETROTRANSPOSON |  |
|  |  |  |  |  | CCHC-TYPE DOMAIN-CONTAINING PROTEIN |  |
|  |  |  |  |  | DUF1985 DOMAIN-CONTAINING PROTEIN |  |
|  |  |  |  |  | TRANSPOSASE, PUTATIVE-RELATED |  |
|  |  |  |  |  | CCHC-TYPE DOMAIN-CONTAINING PROTEIN |  |
|  |  |  |  |  | CCHC-TYPE DOMAIN-CONTAINING PROTEIN |  |
|  |  |  |  |  | CCHC-TYPE DOMAIN-CONTAINING PROTEIN |  |
|  |  |  |  |  | GAG_PRE-INTEGRS DOMAIN-CONTAINING PROTEIN |  |
|  |  |  |  |  | GAG-POL-RELATED RETROTRANSPOSON  (32) |  |
| 2 | *qRTI.2B.2;  qRR.2B.2;  qSSI.2B.2;  qRRG.2B.2* | *2B_1271491–2B_10786746* | 1.27–10.79  (9.52 Mb) | 145.5–146.5 | GATA TRANSCRIPTION FACTOR 18 | Peroxidase 72-Related [80];  GATA Transcription Factor [82]; Cytochrome P450 [82]. |
|  |  |  |  |  | CYTOCHROME P450 84A1-RELATED |  |
|  |  |  |  |  | RED CHLOROPHYLL CATABOLITE REDUCTASE, CHLOROPLASTIC |  |
|  |  |  |  |  | EXPRESSED PROTEIN |  |
|  |  |  |  |  | UNNAMED PRODUCT |  |
|  |  |  |  |  | BETA-GALACTOSIDASE RELATED |  |
|  |  |  |  |  | CDK5 REGULATORY SUBUNIT-ASSOCIATED PROTEIN 1 |  |
|  |  |  |  |  | ZINC FINGER CW-TYPE COILED-COIL DOMAIN PROTEIN 3. |  |
|  |  |  |  |  | PROTEIN DA1-RELATED 2 |  |
|  |  |  |  |  | ZINC FINGER CW-TYPE COILED-COIL DOMAIN PROTEIN 3. |  |
|  |  |  |  |  | G-PROTEIN COUPLED RECEPTOR |  |
|  |  |  |  |  | HYDROXYPROLINE-RICH GLYCOPROTEIN FAMILY PROTEIN-RELATED |  |
|  |  |  |  |  | CRAL-TRIO DOMAIN-CONTAINING PROTEIN YKL091C-RELATED |  |
|  |  |  |  |  | AUXILIN/CYCLIN G-ASSOCIATED KINASE-RELATED |  |
|  |  |  |  |  | UDP-GLYCOSYLTRANSFERASE 72E1 |  |
|  |  |  |  |  | FILAMENT-LIKE PLANT PROTEIN 4 |  |
|  |  |  |  |  | PROTON MYO-INOSITOL COTRANSPORTER |  |
|  |  |  |  |  | METHIONINE AMINOPEPTIDASE |  |
|  |  |  |  |  | ALPHA/BETA-HYDROLASES SUPERFAMILY PROTEIN |  |
|  |  |  |  |  | PEROXIDASE 72-RELATED |  |
|  |  |  |  |  | NUCLEOPORIN P58/P45 NUCLEOPORIN-LIKE PROTEIN 1 |  |
|  |  |  |  |  | GH16843P |  |
|  |  |  |  |  | APOSPORY-ASSOCIATED PROTEIN C-RELATED |  |
|  |  |  |  |  | CYTOPLASMIC TRNA 2-THIOLATION PROTEIN 2 |  |
|  |  |  |  |  | SEVEN TRANSMEMBRANE PROTEIN 1-RELATED |  |
|  |  |  |  |  | CYTOCHROME P450 26 |  |
|  |  |  |  |  | SI:DKEY-122A22.2 |  |
|  |  |  |  |  | BES1/BZR1 HOMOLOG PROTEIN 3-RELATED |  |
|  |  |  |  |  | PROTEIN LATERAL ROOT PRIMORDIUM 1 |  |
|  |  |  |  |  | MYB FAMILY TRANSCRIPTION FACTOR |  |
|  |  |  |  |  | G-BOX-BINDING FACTOR 3-RELATED |  |
|  |  |  |  |  | SI:DKEY-122A22.2 |  |
|  |  |  |  |  | PROTEIN CUP-SHAPED COTYLEDON 2-RELATED |  |
|  |  |  |  |  | TRANSCRIPTION INITIATION FACTOR IIB-RELATED |  |
|  |  |  |  |  | OS03G0411600 PROTEIN |  |
|  |  |  |  |  | HEAT SHOCK TRANSCRIPTION FACTOR |  |
|  |  |  |  |  | UDP-N-ACETYLMURAMOYL-L-ALANYL-D-GLUTAMATE -2, 6-DIAMINOPIMELATE LIGASE |  |
|  |  |  |  |  | ACIDIC LEUCINE-RICH NUCLEAR PHOSPHOPROTEIN 32 |  |
|  |  |  |  |  | U BOX DOMAIN-CONTAINING |  |
|  |  |  |  |  | GATA TRANSCRIPTION FACTOR |  |
|  |  |  |  |  | HOMEOBOX-LEUCINE ZIPPER PROTEIN |  |
|  |  |  |  |  | RNA-BINDING PROTEIN |  |
|  |  |  |  |  | PROTEIN SHROOM-LIKE |  |
|  |  |  |  |  | PLASTID TRANSCRIPTIONALLY ACTIVE PROTEIN |  |
|  |  |  |  |  | PROTEIN SHROOM-LIKE |  |
|  |  |  |  |  | DUF1985 DOMAIN-CONTAINING PROTEIN |  |
|  |  |  |  |  | UBIQUITIN-CONJUGATING ENZYME E2 |  |
|  |  |  |  |  | DUF4228 DOMAIN PROTEIN-RELATED |  |
|  |  |  |  |  | CASP-LIKE PROTEIN 5B3 |  |
|  |  |  |  |  | NUCLEAR AUTOANTIGENIC SPERM PROTEIN NASP -RELATED  (50) |  |
| 3 | *qNF.3B.3* | *3B_6503214–3B_6672912* | 6.50–6.67  (0.17 Mb) | 249.5–250.5 | GLYCOSYL HYDROLASE | Glycosyl hydrolases [83];  E3 ubiquitin-protein ligase RNF13-like [84-85]. |
|  |  |  |  |  | GLYCOSYL HYDROLASE |  |
|  |  |  |  |  | GLYCOSYL HYDROLASE |  |
|  |  |  |  |  | PHYTOL KINASE 1, CHLOROPLASTIC |  |
|  |  |  |  |  | OS07G0183000 PROTEIN-RELATED |  |
|  |  |  |  |  | E3 UBIQUITIN-PROTEIN LIGASE RNF13-LIKE |  |
|  |  |  |  |  | OS07G0183000 PROTEIN-RELATED |  |
|  |  |  |  |  | REVERSE TRANSCRIPTASE ZINC-BINDING  DOMAIN-CONTAINING PROTEIN-RELATED-RELATED  (08) |  |

**Supplementary Table 3.** Genotyping-by-sequencing alignment statistics for all introgression lines (ILs) and parental genotypes shown for sequencing quality assessment

| **SN** | **Genotype** | **Total reads** | **Mapped reads** | **Mapping rate** |
| --- | --- | --- | --- | --- |
| 1 | IL79 | 31,59,130 | 31,18,342 | 98.71% |
| 2 | IL80 | 95,19,640 | 93,84,948 | 98.59% |
| 3 | IL81 | 33,70,908 | 33,26,837 | 98.69% |
| 4 | IL82 | 50,29,892 | 49,55,053 | 98.51% |
| 5 | IL83 | 50,55,078 | 49,91,914 | 98.75% |
| 6 | IL84 | 51,09,658 | 50,16,224 | 98.17% |
| 7 | IL85 | 27,66,000 | 27,23,239 | 98.45% |
| 8 | IL86 | 37,04,830 | 36,51,239 | 98.55% |
| 9 | IL87 | 54,35,076 | 53,66,436 | 98.74% |
| 10 | IL88 | 49,91,060 | 49,26,939 | 98.72% |
| 11 | IL89 | 37,86,324 | 37,48,645 | 99.00% |
| 12 | IL90 | 50,83,052 | 50,30,162 | 98.96% |
| 13 | IL91 | 37,77,104 | 37,23,226 | 98.57% |
| 14 | IL92 | 38,58,370 | 37,95,671 | 98.37% |
| 15 | IL93 | 40,23,542 | 39,41,787 | 97.97% |
| 16 | IL94 | 50,95,760 | 49,75,138 | 97.63% |
| 17 | IL95 | 13,25,918 | 12,88,282 | 97.16% |
| 18 | IL96 | 20,99,586 | 20,74,003 | 98.78% |
| 19 | IL97 | 50,91,528 | 50,33,410 | 98.86% |
| 20 | IL98 | 14,40,236 | 14,09,572 | 97.87% |
| 21 | IL99 | 13,14,286 | 12,94,008 | 98.46% |
| 22 | IL100 | 13,35,170 | 13,17,259 | 98.66% |
| 23 | IL101 | 3,96,010 | 3,82,892 | 96.69% |
| 24 | IL102 | 12,73,286 | 12,60,006 | 98.96% |
| 25 | IL103 | 13,40,672 | 13,28,225 | 99.07% |
| 26 | IL104 | 13,24,826 | 13,08,091 | 98.74% |
| 27 | IL105 | 13,15,892 | 13,01,009 | 98.87% |
| 28 | IL106 | 13,69,624 | 13,53,935 | 98.85% |
| 29 | IL107 | 13,25,484 | 12,72,964 | 96.04% |
| 30 | IL108 | 13,31,514 | 13,18,801 | 99.05% |
| 31 | IL109 | 1,85,25,816 | 1,82,92,101 | 98.74% |
| 32 | IL110 | 25,28,434 | 24,91,667 | 98.55% |
| 33 | IL111 | 89,40,932 | 88,48,378 | 98.96% |
| 34 | IL112 | 85,82,720 | 84,90,647 | 98.93% |
| 35 | IL113 | 14,91,534 | 14,74,798 | 98.88% |
| 36 | IL114 | 14,48,888 | 14,33,700 | 98.95% |
| 37 | IL115 | 37,54,018 | 37,15,668 | 98.98% |
| 38 | IL116 | 1,15,29,158 | 1,13,70,440 | 98.62% |
| 39 | IL117 | 74,18,240 | 73,33,215 | 98.85% |
| 40 | IL118 | 78,62,910 | 77,67,816 | 98.79% |
| 41 | IL119 | 81,45,762 | 80,60,272 | 98.95% |
| 42 | IL120 | 14,50,470 | 14,32,635 | 98.77% |
| 43 | IL121 | 1,20,81,072 | 1,19,36,613 | 98.80% |
| 44 | IL122 | 5,96,178 | 5,78,600 | 97.05% |
| 45 | IL123 | 62,89,862 | 62,22,674 | 98.93% |
| 46 | IL124 | 20,99,566 | 20,74,113 | 98.78% |
| 47 | IL126 | 1,05,59,888 | 1,04,34,967 | 98.82% |
| 48 | IL127 | 54,94,640 | 54,29,156 | 98.81% |
| 49 | IL128 | 48,35,642 | 47,57,378 | 98.38% |
| 50 | IL129 | 13,17,282 | 12,94,176 | 98.25% |
| 51 | IL130 | 13,00,480 | 12,85,586 | 98.85% |
| 52 | IL131 | 13,31,330 | 13,10,835 | 98.46% |
| 53 | IL132 | 75,47,822 | 74,29,851 | 98.44% |
| 54 | IL133 | 65,53,922 | 64,52,889 | 98.46% |
| 55 | IL134 | 39,73,096 | 38,97,505 | 98.10% |
| 56 | IL135 | 30,60,876 | 30,17,476 | 98.58% |
| 57 | IL136 | 1,21,88,724 | 1,20,26,993 | 98.67% |
| 58 | IL137 | 89,25,756 | 88,15,290 | 98.76% |
| 59 | IL138 | 13,31,126 | 13,02,500 | 97.85% |
| 60 | IL139 | 13,28,134 | 13,09,571 | 98.60% |
| 61 | IL140 | 43,52,720 | 43,03,346 | 98.87% |
| 62 | IL141 | 24,75,772 | 24,47,592 | 98.86% |
| 63 | IL142 | 42,63,712 | 41,95,323 | 98.40% |
| 64 | IL143 | 55,99,218 | 55,19,752 | 98.58% |
| 65 | IL144 | 13,18,690 | 12,98,139 | 98.44% |
| 66 | IL145 | 63,21,908 | 62,40,068 | 98.71% |
| 67 | IL146 | 14,12,050 | 13,87,241 | 98.24% |
| 68 | IL147 | 60,34,786 | 59,63,890 | 98.83% |
| 69 | IL149 | 54,98,842 | 53,63,416 | 97.54% |
| 70 | IL151 | 54,73,520 | 53,97,655 | 98.61% |
| 71 | IL152 | 31,46,620 | 30,76,307 | 97.77% |
| 72 | IL153 | 1,01,42,498 | 1,00,13,495 | 98.73% |
| 73 | IL154 | 14,00,046 | 13,83,002 | 98.78% |
| 74 | IL155 | 13,20,662 | 13,05,671 | 98.86% |
| 75 | IL156 | 43,53,876 | 42,97,571 | 98.71% |
| 76 | IL157 | 66,14,390 | 64,26,806 | 97.16% |
| 77 | IL159 | 76,14,876 | 74,94,554 | 98.42% |
| 78 | IL160 | 70,21,530 | 66,69,724 | 94.89% |
| 79 | IL161 | 75,63,564 | 71,82,052 | 98.62% |
| 80 | IL163 | 71,85,400 | 68,52,250 | 98.15% |
| 81 | BC4 | 30,50,012 | 28,85,184 | 94.60% |
| 82 | DRMRIJ-31 | 36,04,624 | 35,60,713 | 98.78% |

**Supplementary Figure 1.** Pearson’s correlation matrix among root architectural traits and stress tolerance indices under aluminium toxicity stress conditions
